# Photoinduced ROS Production by Poly(3‐hexylthiophene) (P3HT) is due to Autoxidation Rather Than Catalysis

**DOI:** 10.1002/chem.71488

**Published:** 2026-07-28

**Authors:** Roman Gańczarczyk, Renata Rybakiewicz‐Sekita, Eric D. Głowacki

**Affiliations:** ^1^ Faculty of Chemistry Warsaw University of Technology Noakowskiego 3 Warsaw Poland; ^2^ Bioelectronics Materials and Devices Laboratory, Central European Institute of Technology Brno University of Technology Purkyňova 123 Brno Czech Republic; ^3^ Faculty of Mathematics and Natural Sciences. School of Exact Sciences Cardinal Stefan Wyszynski University Wóycickiego 1/3 Warsaw Poland

**Keywords:** hydrogen peroxide, nanoparticles, P3HT, photochemistry, photodegradation

## Abstract

Poly(3‐hexylthiophene) (P3HT) is widely reported to generate reactive oxygen species (ROS) under illumination and is often implicitly treated as a photocatalyst in aqueous and biological environments. Here, we demonstrate that ROS formation by P3HT proceeds at the expense of irreversible polymer photooxidation. Systematic irradiation studies of P3HT thin films and nanoparticles under controlled photon flux reveal a strong wavelength dependence of both hydrogen peroxide generation and polymer degradation, with higher photon energies accelerating both processes. Quantitative analysis shows that H_2_O_2_ formation is coupled to loss of conjugation and polymer consumption, and is not suppressed by sacrificial donors such as formate or glutathione. Comparative studies of different types of P3HTs indicate that increased interfacial accessibility as well as low regioregularity enhance degradation, and thus also ROS production. Together, these findings demonstrate that photoexcited P3HT acts as an efficient photosubstrate rather than a true photocatalyst, undergoing self‐sacrificial autoxidation during light‐driven oxygen reduction. This distinction is critical for interpreting ROS‐related phenomena in emerging biological applications.

## Graphical abstract

P3HT is widely treated as a photocatalyst that generates ROS under light. We show otherwise: ROS production comes at the expense of the polymer itself. Photoexcited P3HT acts as a self‐sacrificial photosubstrate, undergoing irreversible autoxidation during light‐driven oxygen reduction—a distinction critical for interpreting its behavior in emerging bioelectronic and biological applications.

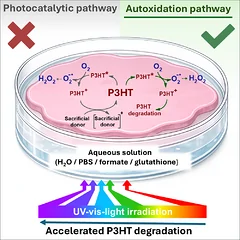

## 1 Introduction

The use of organic semiconductors as photo(electro)stimulation agents for biomodulation applications is an active field of study [1]. Organic semiconductors have outstanding absorbance coefficients, meaning they can efficiently absorb light and then transduce this energy into electrical or chemical potential. This can be used to photostimulate single cells and tissues, for applications ranging from neuromodulation to tissue engineering [1]. One of the most popular organic semiconductors in these contexts is P3HT. Emerging from an established history in polymer solar cells, P3HT is easily obtained and well‐studied [2]. P3HT can be deployed in thin‐film or colloidal‐particle form in a wide range of photostimulation applications, the majority of which exploit the ability of photoexcited P3HT to reduce dissolved oxygen to superoxide and subsequently to the stable reactive oxygen species (ROS) hydrogen peroxide (H_2_O_2_) [2, 3, 4, 5, 6, 7, 8, 9, 10, 11, 12]. Thus, P3HT acts as a reliable photobiomodulator via ROS production. The impressive list of applications of this concept invites the question: What is the full mechanism of ROS photoproduction? While several studies have provided strong evidence that P3HT reduces oxygen via a 1‐electron process to superoxide [13, 14], the unresolved issue is the anodic couple of this redox reaction, that is, what is being oxidized to source the electrons to sustain the photogeneration of ROS. There are two possible pathways, or a coexistence of the two (Figure 1a): (1) A photocatalytic redox cycle: The photogenerated hole in the P3HT polymer may oxidize various electron‐donor compounds, and many biological solutions indeed contain easily oxidizable organic substrates like glutathione or organic acids, which can serve as the source of electrons. (2) An autoxidation process: The photogenerated hole in P3HT leads to irreversible oxidative breakdown of the P3HT itself. The autoxidation mechanism of ROS production is implicated in other biologically relevant situations, notably photogeneration of ROS by the biopigment melanin [15]. This manuscript tests these two hypotheses and provides evidence that P3HT generates ROS via autoxidation (Pathway 2), with no evidence supporting a contribution from Pathway 1.

**FIGURE 1:**
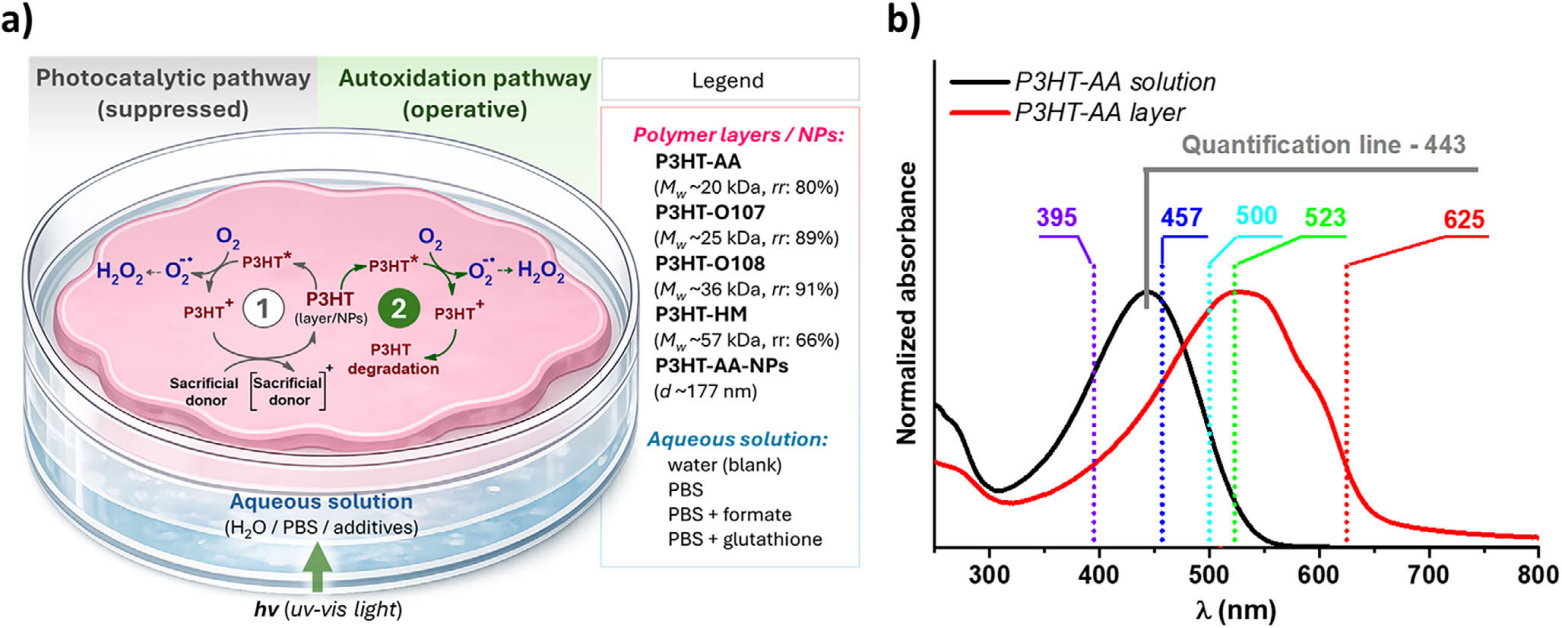
(a) ROS generation by photogenerated excitons on P3HT. Photoreduction of O_2_ proceeds with concurrent oxidation of donors (Sacrificial donor→Sacrificial donor^+^), P3HT itself (autoxidation), or both. By measuring the quantity of evolved H_2_O_2_ and spectrophotometrically quantifying the degradation of P3HT itself, we establish the balance between these two pathways. (b) UV‐vis spectra of the P3HT‐AA in chloroform solution (black line) and thin film deposited on quartz (red line). The dashed lines mark selected wavelengths at which photochemical tests of the P3HT layers were carried out. Quantification of P3HT‐AA content is done on solutions, which obey the Lambert‐Beer law, at 443 nm.

The photostability of P3HT remains one of the key issues in the context of its applications in organic electronics and optoelectronic devices, as reflected in numerous literature reports devoted both to degradation kinetics and to the identification of pathways leading to the loss of optical and electronic properties [16, 17]. In parallel, mechanistic models have been developed to elucidate the nature of the processes responsible for the photodegradation of this polymer (Figure 2).

**FIGURE 2:**
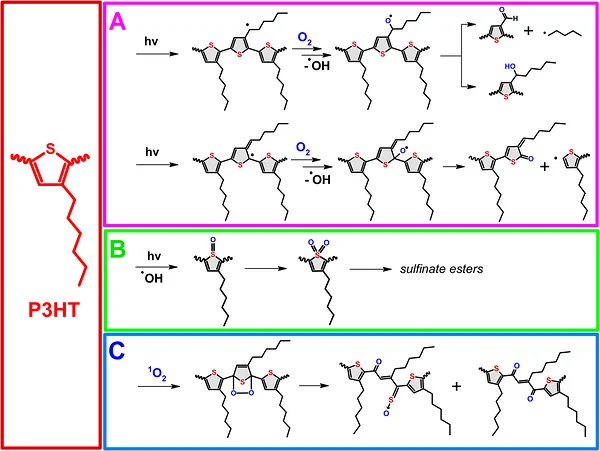
Schematic of the possible photodegradation pathways of P3HT.

One of the most frequently cited scenarios involves P3HT photodegradation occurring through a radical formation at the α‐carbon atom of the hexyl side chain, leading to hydrogen abstraction during light irradiation. These radicals react with oxygen to form peroxides and hydroperoxides, whose photolytic decomposition into alkoxyl radicals results in chain scission (with the formation of ketones and thienyl radicals) or in the generation of aldehydes, secondary alcohols, or cross‐linked structures [18, 19, 20, 21, 22] (Photodegradation Mechanism A, Figure 2). Importantly, Aoyama et al. [23] demonstrated that even in the case of polythiophene derivatives bearing nonalkyl substituents at the 3‐position (e.g., phenyl or *tert*‐pentyl groups), the degradation mechanism proceeds analogously to that observed for P3HT, indicating the dominant role of the thiophene ring in the photo‐oxidation process.

Another frequently described process is the sequential oxidation of the sulfur atom within the thiophene ring (S → sulfoxide → sulfone → sulfinate ester). The identification of sulfinate esters as final degradation products unequivocally indicates cleavage of the thiophene ring and irreversible disruption of the π‐conjugation [18, 24] (Photodegradation Mechanism B, Figure 2).

A singlet‐oxygen‐mediated pathway has also been proposed for P3HT photooxidation, involving 1,4‐addition to the thiophene ring and formation of an unstable endoperoxide intermediate (thiaozonide), which may further decompose into sulfine and trans‐diketone products, leading to oxidation and cleavage of the thiophene ring (Mechanism C, Figure 2) [19, 25]. However, this mechanism should be treated with caution, as Manceau et al. reported that singlet oxygen is not significantly involved in the solid‐state photochemistry of P3HT, suggesting that this pathway may not be a dominant degradation route under typical photooxidative conditions [24].

An important element of the early stage of degradation is also the formation of a reversible charge‐transfer complex between P3HT and molecular oxygen. The formation of such a complex (CTC) results in a transient modulation of the optoelectronic properties of the material and may represent a step preceding irreversible oxidative processes [26].

It is worth noting, however, that the degradation models developed to date have focused primarily on classical photo‐ and thermo‐oxidative pathways. In the context of P3HT, the possibility of photodegradation initiated by autoxidation associated with the presence of a residual hole remaining in the polymer chain as a result of exciton dissociation has not been analyzed in detail. Such long‐lived localization of positive charge may promote oxygen reduction and the generation of hydrogen peroxide. Furthermore, the vast majority of studies on P3HT photodegradation have been conducted in solid‐state systems (thin films) or in organic solutions containing dissolved oxygen, whereas aqueous environments have practically not been the subject of classical mechanistic analyses. An exception is the work of González‐Juárez et al. [27], in which P3HT was investigated in an aqueous system, however, that study focused primarily on colloidal stability and photocatalytic applications rather than on detailed identification of degradation pathways.

The influence of humidity on P3HT photodegradation was systematically analyzed by Hintz et al. [20], who demonstrated that increasing humidity significantly accelerates photo‐oxidation, but only in the presence of oxygen, indicating that water is not a direct reagent initiating polymer decomposition.

Moreover, no reports have been identified that provide a detailed analysis of the photostability of P3HT nanoparticles in aqueous environments, which is of particular importance in the context of emerging biological and biointerfacial research. Considering the growing interest in the application of P3HT in bioelectronic systems, this issue requires in‐depth and systematic investigation.

## 2 Results and Discussion

### 2.1 Preparation and Irradiation Procedure of P3HT Films and NPs

In this work, we tested five different P3HT sample types. Four polymers were investigated as thin films, including three commercially available materials: P3HT‐AA from Alfa Aesar, P3HT‐O107 and P3HT‐O108 from Osilla, and a home‐made polymer, P3HT‐HM. The fifth sample type consisted of aqueous suspensions of P3HT‐AA nanoparticles (P3HT‐AA‐NPs). Both noncommercial sample types were prepared according to literature‐established procedures (P3HT‐HM [12, 28], NPs [4, 5, 12, 29, 30, 31]), with selected modifications introduced to optimize P3HT purification as well as to improve reproducibility and control over nanoparticle size. Detailed experimental protocols for the synthesis, purification, and fractionation of FeCl_3_ ‐ polymerized P3HT, as well as for the preparation and size‐selected isolation of P3HT‐AA nanoparticles, are provided in the subsequent sections of the Supporting Information, Subsections 1.1.1 and 1.1.2. Characterization of all polymers, including ^1^H and ^13^C NMR spectra (Figures S12‐S15), UV‐Vis spectra of solutions and thin films (Figure S11), as well as measurements of nanoparticle size distribution and ζ‐potential (Figure S1), is provided in the Supporting Information.

For P3HT‐AA‐NPs, the final supernatant obtained after differential centrifugation was selected for irradiation and photochemical studies. This fraction contained nanoparticles with an average diameter of 177 ± 41 nm, placing it within the size range representative of P3HT‐based aqueous dispersions previously used in biointerfacial and photostimulation studies, including cellular systems and in vivo light‐transduction experiments [4, 5, 12, 29, 30]. The selection of this fraction was further supported by its stable colloidal character: the negative ζ potential of ‐32.0 ± 8.75 mV indicates good colloidal stability and a reduced tendency toward aggregation, which is essential for maintaining reproducible nanoparticle‐water interfaces during irradiation.

After preparation of the P3HT polymer solutions and P3HT‐AA nanoparticle suspensions, the samples were processed according to a unified irradiation and post‐irradiation analysis workflow (Supporting Information, Sections 2 and 3). For thin‐film P3HT experiments (Supporting Information, Subsection 2.1), PET discs were cleaned, coated with P3HT by drop‐casting, and dried under vacuum. For each P3HT polymer, an independent measurement set consisting of five separate P3HT‐coated PET discs was prepared. Drop‐casting was intentionally used instead of spin‐coating to ensure deposition of a defined and comparable amount of polymer on each substrate (300 ± 30 nmol), which was essential for quantitative post‐irradiation analysis of the residual P3HT after redissolution in chloroform. Film thickness was also considered as a parameter potentially affecting P3HT degradation and hydrogen peroxide production; therefore, the thicknesses of the drop‐cast P3HT films were measured by profilometry, and the corresponding values are provided in the Supporting Information (Table S1). Although drop‐cast films showed thickness variations and lower uniformity, the photodegradation and hydrogen peroxide generation data obtained for the three regioregular polymers, P3HT‐AA, P3HT‐O107, and P3HT‐O108, were very similar, indicating that film thickness variability did not dominate the observed photochemical trends.

Each measurement set used in the photochemical experiments included one nonirradiated reference disc used to determine the initial amount of deposited P3HT and four test discs, which were placed polymer‐side down in oxygenated aqueous media: deionized water, phosphate‐buffered saline (PBS), PBS containing sodium formate, or PBS containing reduced glutathione (GSH). The samples were irradiated for 15 h under controlled light power density (Table 1). To minimize the contribution of thermally induced degradation, all samples were continuously cooled during irradiation, and the temperature of the solutions did not exceed 27°C. For the P3HT‐AA nanoparticle experiments, the aqueous P3HT‐AA‐NP suspension was mixed with formate‐containing phosphate buffer, divided into individual aliquots, and irradiated under green light (523 nm) for 15 h, as described in the Supporting Information, Subsection 2.2.

**TABLE 1:** Radiation power density values and photon fluxes for the indicated wavelengths.

*λ* (nm)	*P* (mW∙mm*^−2^*)	*Φ (nEs*∙*s^−1^mm^−2^)*
395	0.153	0.505
457	0.148	0.564
500	0.153	0.640
523	0.154	0.671
625	0.148	0.773

### 2.2 Quantifying H_2_O_2_ Generation and P3HT Degradation

The photochemical experiments on P3HT samples were carried out as schematized in Figure 1a. To evaluate the relationship between hydrogen peroxide photogeneration and polymer degradation, both the concentration of generated H_2_O_2_ and molar amount of P3HT were determined using spectrophotometric methods (Figure 2b).

After irradiation, P3HT films and P3HT‐AA nanoparticles were processed according to separate but analogous workflows, detailed in the Supporting Information, Section 3. For P3HT thin films, the irradiated PET discs (Figure S9) were removed from the aqueous media, rinsed, dried, and the remaining P3HT layer was dissolved in chloroform for UV–Vis‐based quantification of polymer degradation (Figure S6). The corresponding aqueous solutions were used for hydrogen peroxide determination (Figure S6). For nanoparticles, aliquots of the irradiated suspensions were directly used for hydrogen peroxide quantification (Figure S7), whereas the remaining suspension was dried, redissolved in chloroform, and analyzed by UV–Vis spectroscopy to determine the residual P3HT content (Figure S7). These procedures enabled direct comparison between hydrogen peroxide generation and P3HT degradation for both sample formats. To quantify the contributions of Pathways 1 and 2 to photogeneration of H_2_O_2_ on P3HT, two figures of merit can be computed. The *k* parameter is defined as the ratio of moles of evolved H_2_O_2_ to the initial number of moles of P3HT repeat units initially used, while the degradation ratio corresponds to the ratio of moles of P3HT consumed relative to its initial amount It should be specified that polymer degradation was quantified from the residual amount of P3HT after redissolution in chloroform, using calibration curves prepared for each P3HT sample from standard chloroform solutions. In turn, hydrogen peroxide was quantified using the HRP/TMB assay [32], in which H_2_O_2_ oxidizes TMB in the presence of horseradish peroxidase to form a blue chromophore absorbing at 653 nm. H_2_O_2_ concentrations were calculated from a calibration curve prepared from standard hydrogen peroxide solutions. Detailed descriptions of the photocatalytic test methodology, including photoreactor power calibration, preparation of P3HT calibration curves used to determine the degree of degradation, and spectrophotometric determination of H_2_O_2_ concentration using the HRP/TMB method, are provided in the Supporting Information (Section 3).

As previously mentioned, the photochemical experiments were carried out under controlled irradiation conditions (excitation wavelength and power). The light sources were adjusted so that the power (in W/mm^2^) at each wavelength was as similar as possible. Consequently, the photon flux of the applied irradiation light gradually increased with lowering light energy. Detailed conditions are summarized in Table 1. The P3HT films used in this study exhibited absorbance values exceeding 3 across the investigated spectral range (300–650 nm), corresponding to absorption of more than 99.9% of the incident light. Therefore, it can be assumed that all incident photons were absorbed regardless of the excitation wavelength. The results presented in Figure 3 and Table S2 demonstrate a strong dependency of the polymer degradation, hydrogen peroxide concentration, and consequently the *k* values on the photon energy. Notably, despite the lower photon flux at shorter wavelengths, both polymer degradation and H_2_O_2_ generation were substantially enhanced under high‐energy excitation, indicating that photon energy plays a dominant role in determining the efficiency of the photochemical processes. When considering the data and associated errors within each wavelength series, no consistent trend in photostability can be identified with respect to the composition of the aqueous electrolytes (pure water, PBS solution, and PBS containing the donor formate). The fact that formate, a commonly used sacrificial donor, neither suppresses the autodegradation of P3HT nor enhances H_2_O_2_ formation signals that donor oxidation is not part of the redox cycle. We found that the addition of glutathione, an even stronger electron donor and also a potent ROS scavenger, slightly diminishes the extent of P3HT degradation. Not surprisingly, glutathione eliminated detectable [H_2_O_2_]. The slight improvement in P3HT stability upon addition of glutathione can be explained by two mechanisms: either glutathione scavenges holes and thus counteracts autoxidation, or by eliminating resultant hydrogen peroxide, it protects P3HT from secondary oxidative degradation caused by hydrogen peroxide itself. However, the most important result is that, in all cases, the degradation degree is greater than 0 while the parameter *k* remains below 1. This indicates that the material cannot be regarded as a photocatalyst under these conditions. A catalyst should not be consumed during the reaction; therefore, any measurable degradation of the P3HT layer already contradicts catalytic behavior. Moreover, *k* values below 1 indicate that the formation of hydrogen peroxide is sub‐stoichiometric with respect to the degradation of the material. Consequently, photocatalytic mechanisms must be excluded.

**FIGURE 3:**
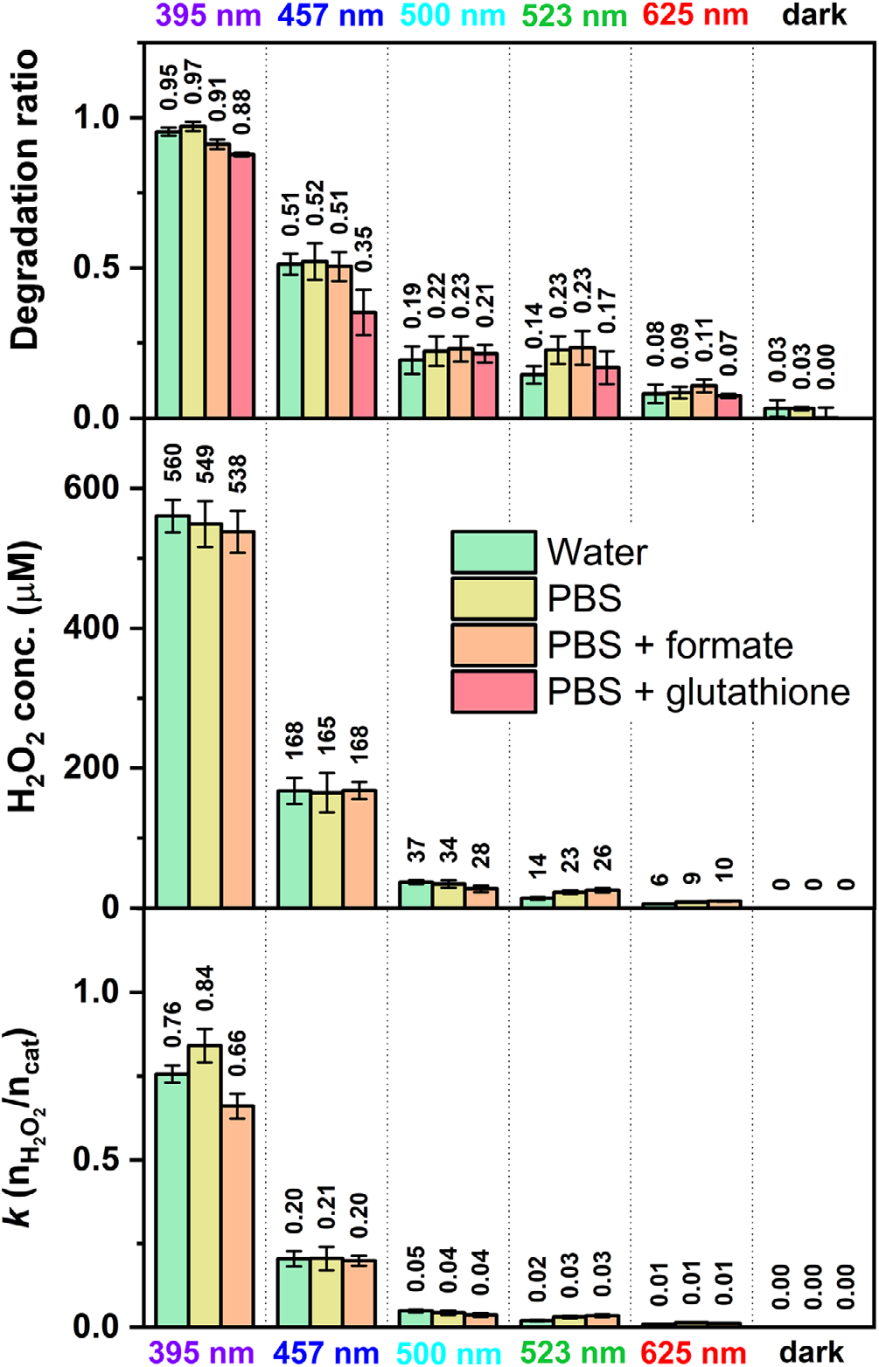
Photochemical production of hydrogen peroxide on P3HT‐AA layers deposited on PET foil under irradiation with light of various wavelengths at 15 mW/cm^2^. Each experiment was conducted for a 15 h irradiation period using 300 ± 30 nmol of P3HT layer drop‐cast on circular PET foils (Ø ≈ 1.5 cm). Solution volumes were 0.4 mL. If added (see legend), the sacrificial donor concentration was 10 mM. In the experiments conducted with glutathione, it was not possible to determine [H_2_O_2_] due to its immediate reduction to water. Error bars represent the standard deviation across n = 4 replicate measurements, each performed on a separately irradiated P3HT sample.

We next turned our attention to photochemically comparing the different forms of P3HT, as we hypothesized that differences in molecular weight or regioregularity will impact both stability and hydrogen peroxide formation (Figure 4 and Table S3). Further, we prepared colloidal P3HT nanoparticles, since this is the most popular form of P3HT used for biological photostimulation experiments. All samples were tested using green light, 523 nm, for the reason that this is the typical irradiation wavelength for biological applications. It is immediately apparent that the colloidal nanoparticles, despite comprising over sixfold lower molar quantity of P3HT, are the most efficient generators of H_2_O_2_. This also corresponds to the highest degree of degradation. This result can be rationalized by pointing out that colloidal nanoparticles have much higher interfacial area (Table S3) for photochemical oxygen reduction to occur, and thus the whole process of autoxidation and accompanying hydrogen peroxide production happens faster. In comparing the thin films of different P3HT types to each other, the degradation degree, hydrogen peroxide concentration and *k* values are statistically the same for P3HT‐AA, P3HT‐O107 and P3HT‐O108. In contrast, P3HT‐HM deviates from this trend, exhibiting more than twice the degradation level and H_2_O_2_ concentration, and an approximately 1.5‐fold higher *k* than the other polymers. This difference can be consistently attributed to the substantially lower regioregularity of P3HT‐HM, as calculated from NMR (SI). In line with the work of Hintz et al. [20], lower regioregularity may increase the triplet‐state formation yield, thereby facilitating photoinduced radical pathways and leading to faster photo‐oxidative degradation.

**FIGURE 4:**
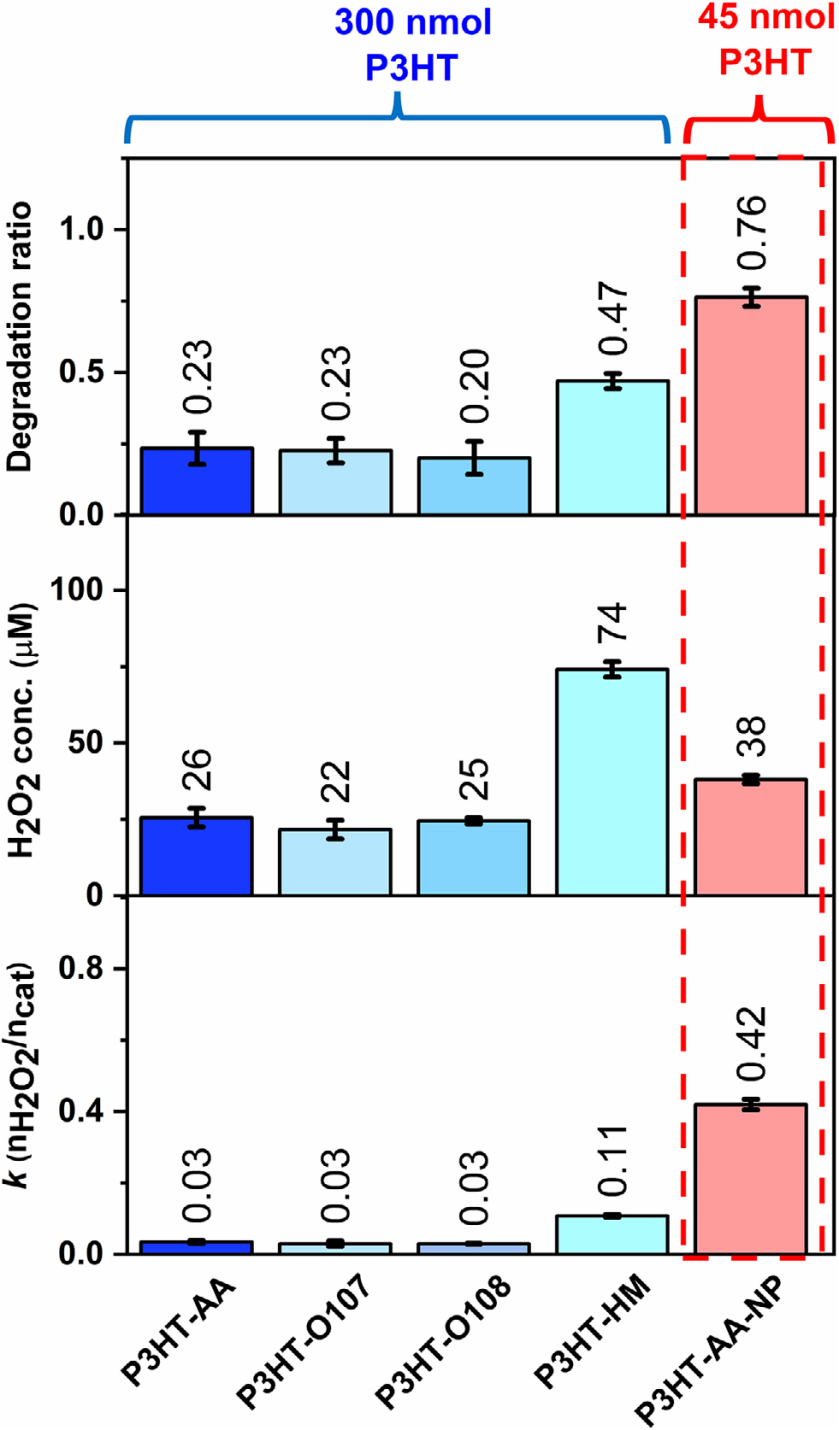
Photochemical tests for four P3HT layers (P3HT‐AA, P3HT‐HM, P3HT‐O107, P3HT‐O108; 300 ± 30 nmol each) and for an aqueous suspension of P3HT nanoparticles (45 nmol, average diameter 177 nm, ζ‐potential −32 mV) prepared from P3HT‐AA. All tests were carried out in 0.4 mL PBS containing formate (10 mM) as sacrificial donor, under 15 h of green‐light irradiation (523 nm, 0.154 mW/mm^2^). The error bars refer to the estimated standard deviation of four measurements performed on separate irradiated P3HT layers/NPs solutions.

## 3 Conclusion

The photoproduction of ROS (and in particular hydrogen peroxide) on P3HT holds great promise in numerous biological applications. Our work was motivated by the desire to understand this process and identify if it is a photochemical autoxidation reaction or indeed a photocatalytic process which can use available antioxidant molecules as fuels. The experiments show that hydrogen peroxide production is strongly correlated with polymer degradation. Together with the finding that the degradation degree is greater than 0 while the parameter *k* remains below unity, this indicates that autoxidation is the dominant pathway rather than photocatalysis. There have been a number of mechanistic studies on different molecular pathways of oxidative P3HT degradation, which we provide in the Introduction section. Our tests of using formate or glutathione as electron donors did not mitigate polymer photodegradation. A limitation of our study is that indeed that other donors were not tested; it is conceivable that donors present in certain biological media may enable some catalytic redox pathway. However, our finding of sub‐stoichiometric hydrogen peroxide production accompanied by P3HT autoxidation shows that this is an important mechanism that should be accounted for in biological deployments of P3HT for photostimulation. Consequently, as the process oxidatively consumes P3HT as a fuel, the photostimulation effect will be time‐limited. Moreover, the interpretation of biological outcomes may be complicated by the impact of degradation products of P3HT. Further work is needed to fully understand the mechanisms behind P3HT photostimulation and possible byproduct effects, and to explore avenues of true photocatalytic organic semiconductor systems which produce hydrogen peroxide while consuming various electron donors.

## Conflicts of Interest

The authors have no conflicts of interest.

## Supporting information




**Supporting File 1**: chem71488‐sup‐0001‐SuppMat.pdf
The Supporting Information includes detailed experimental procedures, the synthesis and preparation of P3HT and its nanoparticles, materials characterization, irradiation protocols, methods for quantifying H_2_O_2_ and polymer degradation (including *k* determination), as well as profilometric measurements of P3HT film thickness. It also contains spectra and supplementary data supporting the main findings. The authors have cited additional references within the Supporting Information [4, 5, 12, 28‐32].

## Data Availability

The data that support the findings of this study are available from the corresponding author upon reasonable request.
