# Supplementary material for: Photoinduced ROS Production by Poly(3‐hexylthiophene) (P3HT) is due to Autoxidation Rather Than Catalysis
Source: Chemistry. 2026 Jul 28;32(40):e71488. doi: 10.1002/chem.71488 (PMC13716019; doi:10.1002/chem.71488)
Supplement: Supplementary file 1 [file CHEM-32-e71488-s001.pdf]

Supporting Information

Photoinduced ROS Production by Poly(3-hexylthiophene) (P3HT) is due to Autoxidation rather than Catalysis

Roman Gańczarczyk[1,2], Renata Rybakiewicz-Sekita[3], Eric D. Głowacki[2]*

[1] Warsaw University of Technology, Faculty of Chemistry, Noakowskiego 3, 00-664, Warsaw, Poland

[2] Bioelectronics Materials and Devices Laboratory, Central European Institute of Technology, Brno University of Technology, Purkyňova 123, Brno 61200, Czech Republic

[3] Cardinal Stefan Wyszynski University, Faculty of Mathematics and Natural Sciences. School of Exact Sciences, Woycickiego 1/3, 01-938, Warsaw, Poland

1. Materials and Characterization techniques 2
   1.1. P3HT samples and reagents 2
   1.1.1. Oxidative polymerization of 3-hexylthiophene using $FeCl_3$ (P3HT-HM) 2
   1.1.2. Preparation and purification of P3HT-AA nanoparticles (P3HT-AA-NPs) 3
2. Preparation and irradiation procedure of P3HT films and NPs 4
   2.1. Preparation and irradiation procedure of P3HT films 4
   2.1.1. Preparation of PET substrates 5
   2.1.2. Deposition of P3HT films 5
   2.1.3. Irradiation procedure for P3HT films 5
   2.2. Preparation and irradiation procedure for P3HT nanoparticles 6
3. Assessment of Material Degradation Extent 7
   3.1. Preparation of the P3HT Calibration Curve 8
   3.2. Hydrogen Peroxide Quantification (HRP/TMB Method) 8
   3.2.1. Preparation of Solutions for H_2O_2 Quantification 9
   3.2.1.1. Citrate–phosphate buffer (pH 5.5 – 6.0) 9
   3.2.1.2. Preparation of the TMB solution (10 mg/mL) 9
   3.2.1.3. Preparation of the HRP solution (2 mg/mL) 9
   3.2.1.4. Preparation of the final reaction mixture for analysis 9
   3.3. Calibration curve for H_2O_2 Determination 9
   3.4. Parameter *k* determination 9
4. Suplementary references 20

1. Materials and Characterization techniques

1.1. P3HT samples and reagents

A total of five poly(3-hexylthiophene-2,5-diyl) (P3HT) samples were used in this study. Three samples were commercially purchased from *Alfa Aesar (AA)* and *Ossila (O)*:

- **P3HT-AA** (Alfa Aesar, batch P10A042),
  Mw = 20000 g·mol^{-1}, PDI = 2.3, regioregularity = 80% (based on ^{1}H NMR (***Fig. S12***)),

- **P3HT-O107** (Ossila, batch M107),
  Mw = 24480 g·mol^{-1}, PDI= 2.8, regioregularity = 89% (based on ^{1}H NMR (***Fig. S13***)),

- **P3HT-O108**, (Ossila, batch M107),
  Mw = 36010 g·mol^{-1}, PDI= 2.7, regioregularity = 91%, (based on ^{1}H NMR (***Fig. S14***)),

The remaining two materials were prepared in-house:

- **P3HT-HM**, synthesized *via* oxidative polymerization using $FeCl_3$ as catalyst[28],
  Mw = 57580 g·mol^{-1} (GPC, DCM, polystyrene standard), PDI = 3.0, regioregularity = 66% (based on ^{1}H NMR (***Fig. S15***)),

- **P3HT-AA-NPs**, a nanoparticle formulation of P3HT-AA produced specifically for this study by nanoprecipitation followed by purification and size fractionation[12],
  $d_{average}$ =177 nm, ζ-potential −32 mV (***Fig. S1***)

3-Hexylthiophene (3HT), iron (III) chloride, horseradish peroxidase (HRP, type I), 3,3′,5,5′-tetramethylbenzidine (TMB), phosphate-buffered saline tablet (PBS), citric acid, sodium and potassium phosphate salts, sodium formate, and glutathione were purchased from Sigma Aldrich and used as received. Hydrogen peroxide (30%), chloroform, methanol and tetrahydrofuran were purchased from Chempur.

1.1.1. Oxidative polymerization of 3-hexylthiophene using $FeCl_3$ (P3HT-HM)

The oxidative chemical polymerization of 3-hexylthiophene was carried out according to a modified $FeCl_3$ protocol[28]. A nitrogen-purged three-neck round-bottom flask was charged with anhydrous chloroform (100 mL). Under a nitrogen atmosphere, a solution of $FeCl_3$ (4 equiv., 3.870 g, 23.859 mmol) in chloroform was added. Subsequently, a solution of 3-hexylthiophene (3HT) (1.000 g, 5.942 mmol) in chloroform (40 mL) was added dropwise over 20 min. The reaction mixture was stirred at room temperature for 20 h. The reaction was then quenched by pouring the mixture into methanol (200 mL), resulting in the precipitation of the crude polymer. Purification was performed by Soxhlet extraction using methanol and acetone (12–24 h each). After drying under vacuum, the black solid was repeatedly rinsed with a 2% (*w/w*) aqueous hydrazine solution. The final solid was dissolved in 200 mL THF, passed through filter, precipitated using 500 mL of methanol and dried under vacuum at 40 °C for two days, yielding 380 mg of P3HT-HM.

The polymer was characterized by ^{1}H and ^{13}C NMR spectroscopy (Bruker 500 MHz, ***Fig. S15***), UV–Vis absorption spectroscopy (Varian Cary 5000, ***Fig. S8***), and gel permeation chromatography (GPC, Viscotek system comprising GPCmax and TDA 305 equipped with DVB Jordi gel column (linear, mixed bed)).

1.1.2. Preparation and purification of P3HT-AA nanoparticles (P3HT-AA-NPs)

P3HT-AA nanoparticles were prepared using a nanoprecipitation method[4,5,12,29–31] (***Fig S5***). P3HT-AA was dissolved in THF at a concentration of 7 mg in 350 μL of solvent. The polymer solution was then rapidly injected dropwise into 7 mL of deionized water (nonsolvent) under continuous stirring at room temperature. The volume ratio of polymer solution to nonsolvent was 1:20. The mixture was stirred for 2 min at 400 rpm.

Removal of residual THF was performed by dialysis. The resulting P3HT-AA-NPs was transferred into dialysis tubing made of regenerated cellulose (Spectra/Por, MWCO 10 000 Da), pre-wetted in 0.05% sodium azide. The dialysis bag was immersed in a large excess of deionized water (approximately 100-fold volume excess). The external water bath was replaced several times over a period of 48 h. During this process THF diffused across the membrane, while P3HT-AA nanoparticles remained inside the dialysis tubing.

Purified P3HT-AA-NPs were subsequently subjected to differential centrifugation to remove large aggregates and obtain size-selected nanoparticle populations. The initial dispersion was centrifuged at 2000 rpm for 10 min at room temperature. The pellet, consisting of large aggregates and mechanical impurities, was discarded, and the supernatant was collected for further processing. Sequential centrifugation steps were then carried out at 4000 rpm, 6000 rpm, 10 000 rpm and 12 000 rpm (10 min each). After each step, the pellet was collected as an individual size fraction, while the supernatant was transferred to the next centrifugation stage.

All collected fractions were characterized by dynamic light scattering (DLS) at 25 °C. ζ potential was additionally determined for each fraction. DLS and ζ potential measurements were performed using a Zetasizer Nano ZS (ZEN3600, Malvern Instruments) equipped with a 633 nm laser illumination.

The final supernatant obtained after centrifugation at 12 000 rpm for 10 min was selected for subsequent irradiation and photochemical studies. This fraction contained nanoparticles with an average diameter of 177 (± 41) nm and a ζ potential of -32.0 (± 8.75) mV (***Fig. S1***). The strongly negative ζ potential values indicate that the P3HT colloids are highly stable.

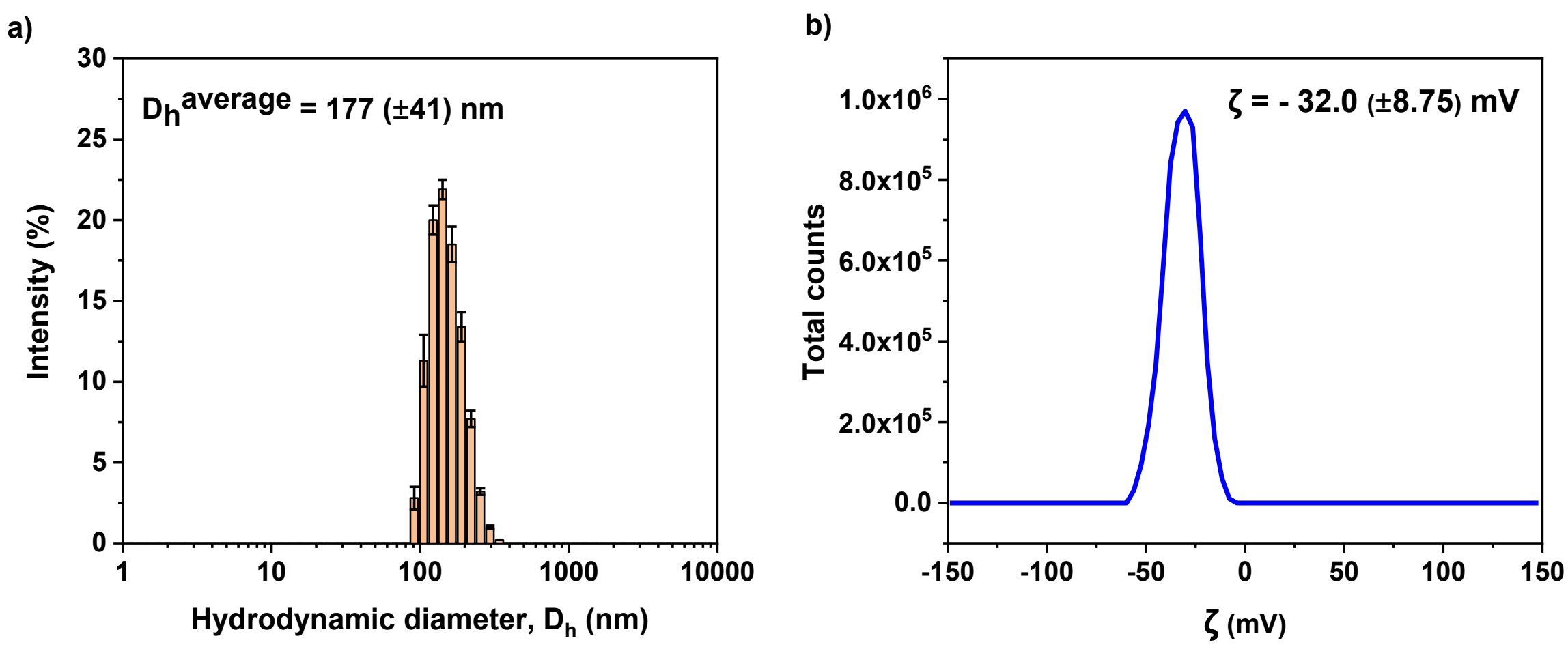

Fig. S1. (a) Particle size distributions (statistics on 3 independent measurements) and (b) ζ potential of P3HT-AA-NPs.

2. Preparation and irradiation procedure of P3HT films and NPs

The procedures described below were applied identically and reproducibly to all investigated samples and consisted of the following sequential stages:

2.1. Preparation and irradiation procedure of P3HT films

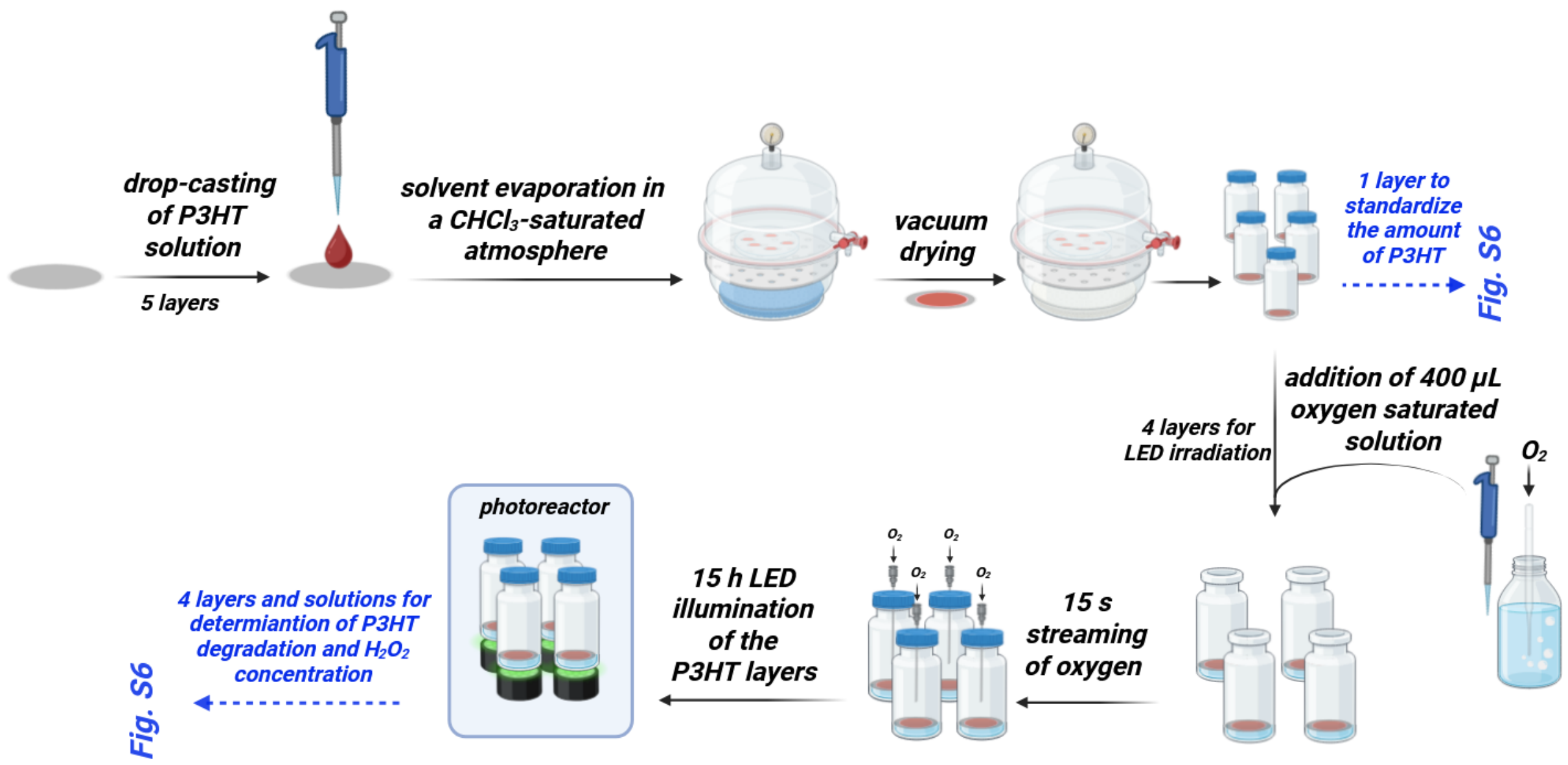

Fig. S2. Scheme of preparation and irradiation procedure of P3HT films. For each P3HT polymer, five separate P3HT-coated PET discs were prepared by drop-casting the polymer solution onto PET substrates, followed by solvent evaporation in a $CHCl_3$-saturated atmosphere and vacuum drying. One disc was kept as a non-irradiated reference sample to determine the initial amount of deposited P3HT, while the remaining four discs were placed polymer-side down in individual vials containing oxygen-saturated aqueous media and irradiated for 15 h under LED illumination. Created with BioRender.com

2.1.1. Preparation of PET substrates

PET substrates in the form of circular discs (12–15 mm in diameter) were cleaned by sequential sonication in isopropanol and deionized water (DI) for 5 min each. After cleaning, substrates were dried under a stream of nitrogen.

2.1.2. Deposition of P3HT films

For each investigated P3HT polymer, five separate PET discs were coated with the corresponding P3HT solution. Solutions of P3HT in chloroform (3.33 mg/ mL) were deposited on the PET discs using the drop-casting method (***Fig. S2, S3***). A volume of 15 µL of the polymer solution was applied to each disc. Solvent evaporation was carried out in a chloroform-saturated atmosphere inside a desiccator to ensure uniform film formation, followed by drying under high vacuum for ~30 min. The average thicknesses of the resulting P3HT layers were determined by profilometry (Bruker Dektak XT) and are summarized in ***Table S1***.

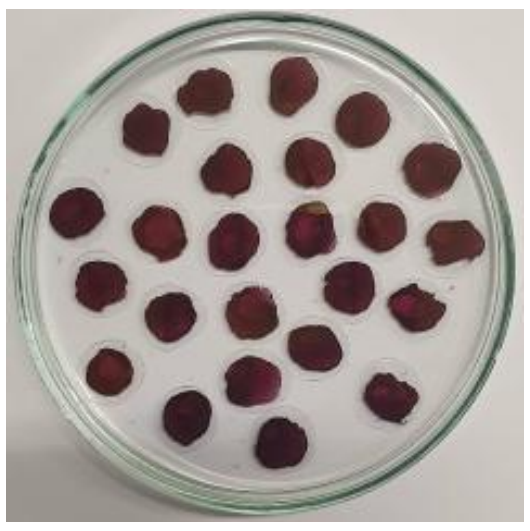

Fig. S3. Photograph of P3HT-AA layers deposited on PET substrates.

Table S1. Thickness of the P3HT layers deposited on PET substrates by drop-casting. The thickness values are reported as mean ± standard deviation based on three independent profilometry measurements.

Layer	*d (nm)*
P3HT-AA	137 (±53)
P3HT-O107	614(±287)
P3HT-O108	501(±163)
P3HT-HM	187(±73)

2.1.3. Irradiation procedure for P3HT films

For each P3HT polymer, one measurement set consisted of independently prepared P3HT-coated PET discs. One disc was kept as a non-irradiated reference sample and used to determine the initial amount of deposited P3HT spectroscopically. The remaining four discs were used as test samples and transferred polymer-side down into individual glass vials (***Fig S2, S4***).

Each of four vials was filled with 400 µL of one of the following oxygenated aqueous media:

a) deionized water (H_2O),

b) phosphate-buffered saline (PBS),

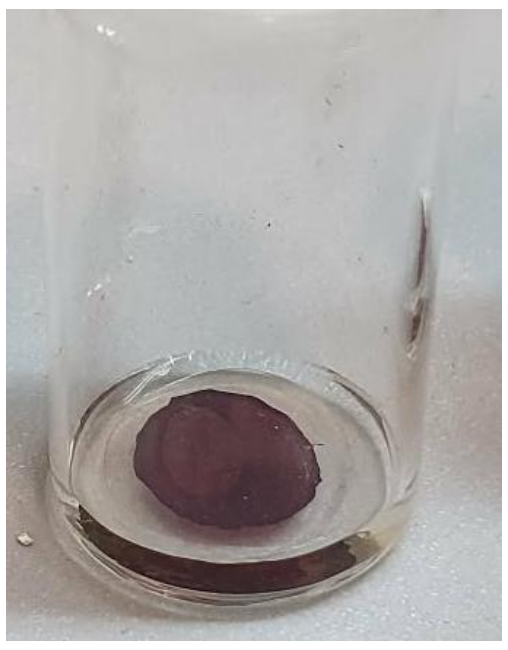

Fig. S4. Photograph of P3HT-AA layer on a PET disc placed in a vial with an aqueous solution.

c) PBS containing 10 mM sodium formate (HCOONa),

d) PBS containing 10 mM reduced glutathione (GSH).

Prior to withdrawing 400 µL, the solution was saturated with oxygen by bubbling for 15 min immediately before the measurements. Positioning the polymer layer downward ensured maximal contact of the active film surface with the oxygenated aqueous medium. Additionally, after loading the PET discs and solutions into each vial, a brief (15 s) stream of oxygen was applied above the PET discs. The vials were then placed in the sample holder of a photoreactor (PhotoCube, ThalesNano) and irradiated for 15 h. The incident light power density was adjusted using a power meter (Thorlabs PM100D) equipped with photodiode sensors (S120VC and S121C, Thorlabs), and the set values are presented in ***Table 1*** of the main text. To minimize the contribution of thermally induced degradation processes during irradiation, the samples were cooled using a continuous flow of cold air generated by a fan equipped with a Peltier cooling module. The cooling system was directed around the sample vials throughout the irradiation experiments. Under these conditions, the temperature of the irradiated solutions did not exceed 27 °C. The temperature was monitored using both a contact thermometer and an infrared pyrometer.

After irradiation, the discs were removed, rinsed thoroughly with DI water, and dried under a stream of nitrogen.

2.2. Preparation and irradiation procedure for P3HT nanoparticles

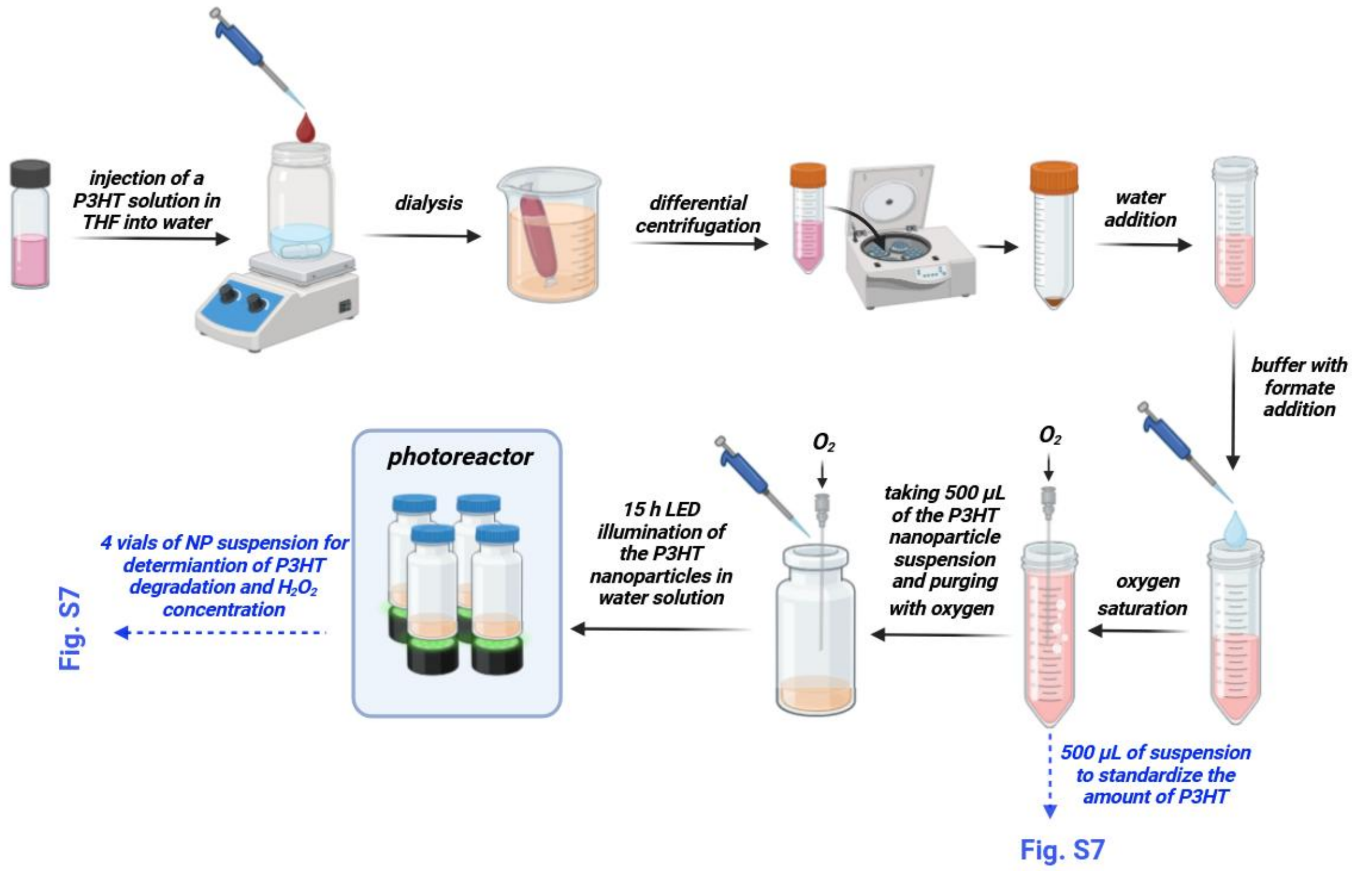

Fig S5. Scheme of preparation and irradiation procedure of P3HT-AA-NPs. Created with BioRender.com.

2200 µL of the aqueous P3HT-AA-NPs suspension was combined with 550 µL of a 50 mM formate solution in PBS buffer and mixed thoroughly (***Fig. S5***). A NaCl- and KCl-free phosphate buffer was used to prevent ionic-strength-dependent aggregation. The buffer was prepared by dissolving 2.38 g Na_2HPO_4 and 0.19 g KH_2PO_4 in a 1 L volumetric flask and adjusting the pH to 7.4. Four aliquots of 500 µL each were then taken from the mixture and subjected to green-light irradiation for 15 hours.

From this point onward, the work on P3HT films samples proceeded along two parallel pathways. The irradiated P3HT films (*Fig. S6, S9, S11*) were used to determine the extent of polymer degradation, whereas the solutions collected from above the irradiated samples were employed to quantify the concentration of the generated hydrogen peroxide (*Fig. S6*), as described in the sections below.

3. Assessment of Material Degradation Extent

P3HT layers (***Fig. S6***)**:** Firstly, each disc was then transferred polymer-side up into a clean vial, filled with 2.5 mL chloroform, and subjected to sonication for 15 min to fully dissolve the P3HT layer for subsequent quantitative analysis. The vials containing the P3HT films were weighed before and after solvent addition to accurately determine the volume of chloroform present. The resulting P3HT solutions were transferred to quartz cuvettes, and their UV–Vis spectra were recorded. Polymer degradation was quantified using the Lambert-Beer law by determining the amount of residual P3HT after dissolving the irradiated layers in chloroform, based on a previously established calibration curve (***Fig. S6***).

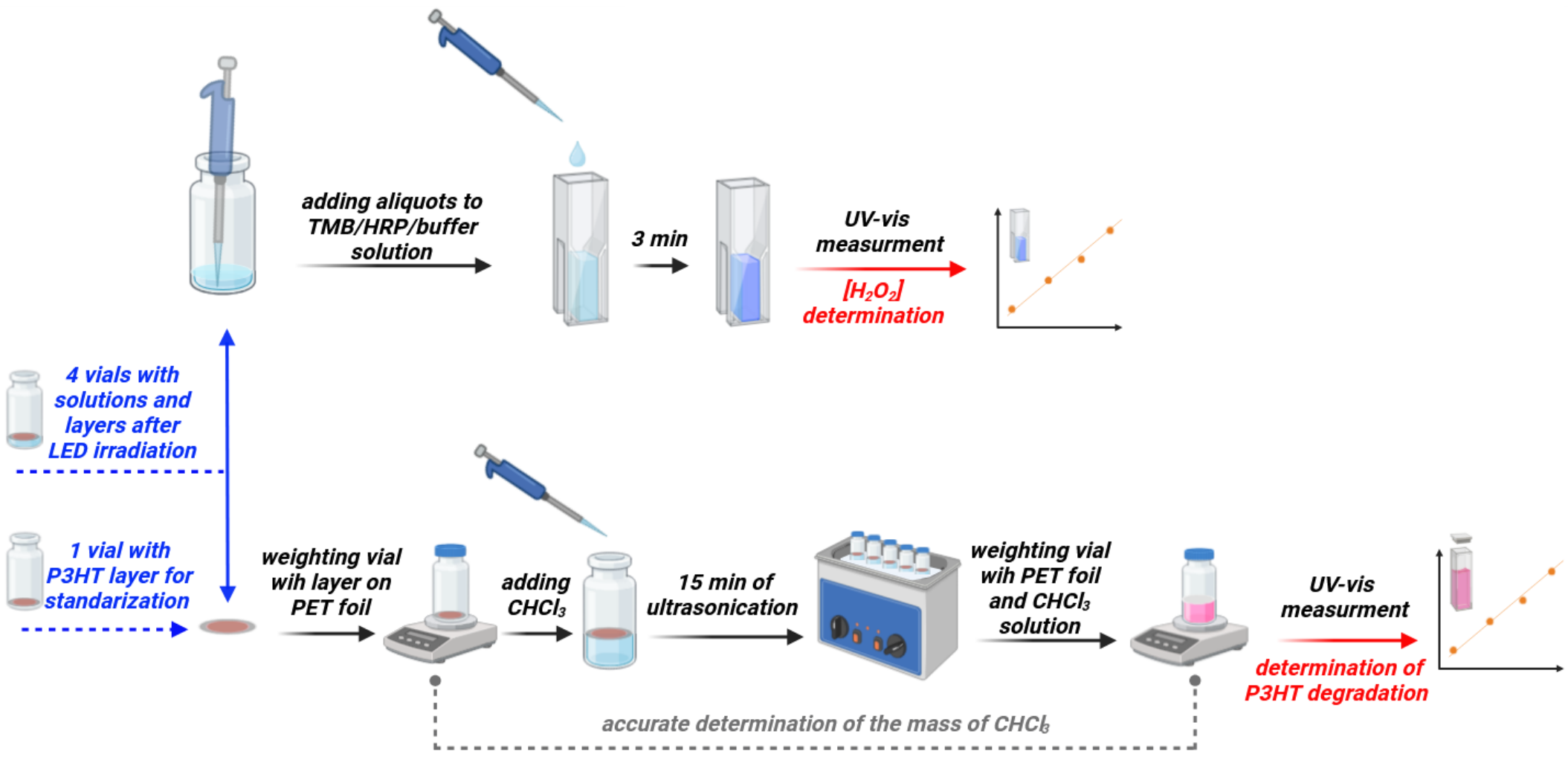

Fig. S6. Scheme of the assessment of P3HT layer degradation extent and determination of the produced H_2O_2. Created with BioRender.com.

P3HT nanoparticles (Fig. S7)**:** After irradiation, 100 µL from each sample was withdrawn, brought to a final volume of 1.00 mL by adding a freshly prepared HRP/TMB reagent mixture in citrate–phosphate buffer, and subsequently used for H_2O_2 quantification. The remaining portions of the irradiated nanoparticle suspensions were evaporated to dryness for subsequent analysis of the degree of polymer NPs degradation. The vials containing the P3HT nanoparticles were weighed before and after solvent addition to accurately determine the volume of

chloroform present. The resulting P3HT solutions were transferred to quartz cuvettes, and their UV–Vis spectra were recorded. Polymer degradation was quantified using the same procedure as that applied for the polymer layers. (***Fig. S7***)

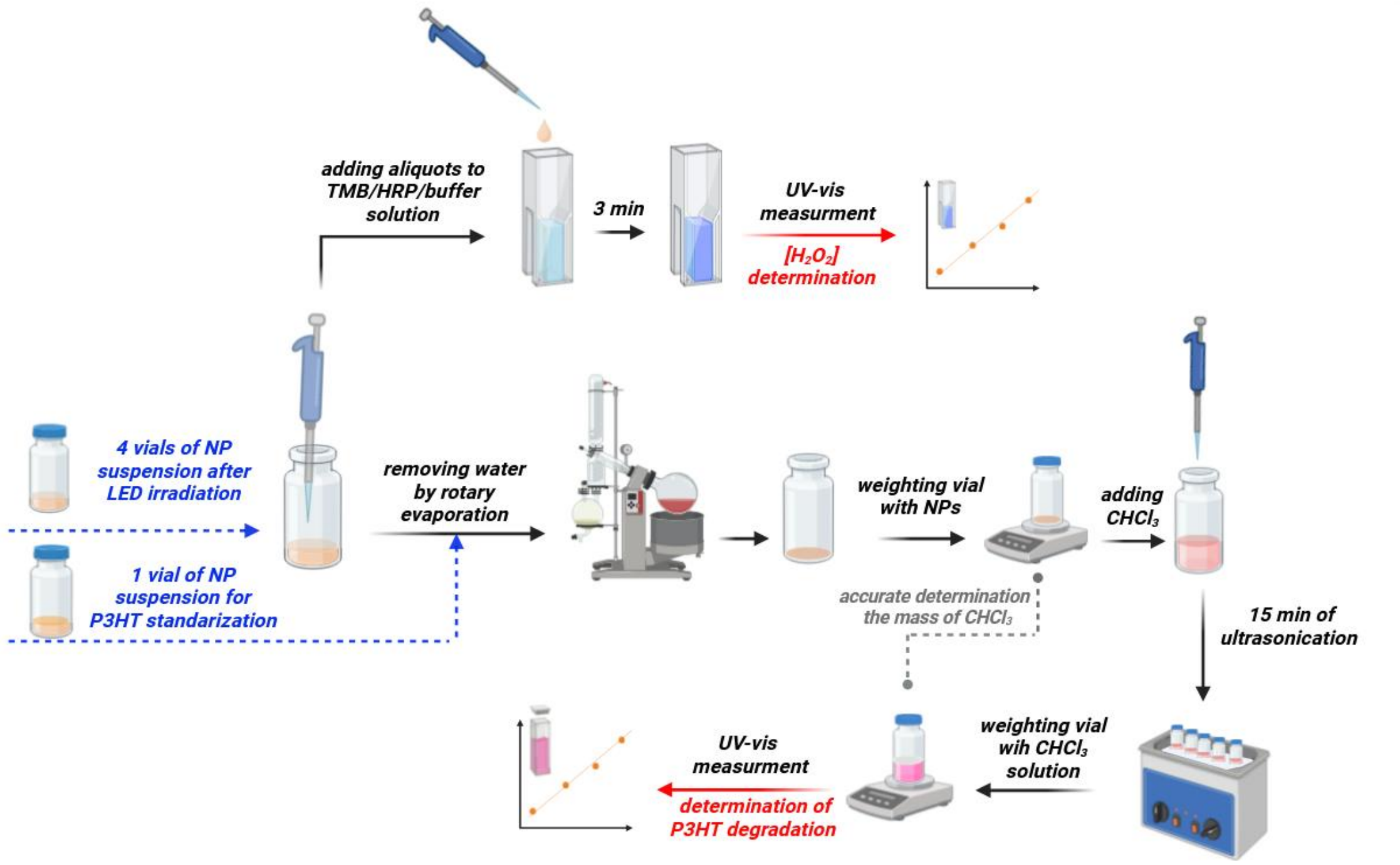

Fig. S7. Scheme of the assessment of P3HT-AA-NPs degradation extent and determination of the produced H_2O_2. Created with BioRender.com.

3.1. Preparation of the P3HT Calibration Curve

The calibration curve was prepared for each P3HT (AA, O107, O108, HM) using a series of standard P3HT solutions in chloroform. First, polymer solutions were prepared at a concentration of 12.5 mg in 50 mL of chloroform. After complete dissolution of P3HT samples the calibration curve were prepared. Specifically, the following volumes of the P3HT solution were transferred into the measurement cuvette: 10 µL, 25 µL, 50 µL, 100 µL, 175 µL, 250 µL, and 500 µL, and then each sample was filled up with chloroform to a final volume of 2.5 mL. For each sample, the absorbance was measured at the maximum of the respective polymer: 443 nm for P3HT-AA, 431 nm for P3HT-HM, 450 nm for P3HT-O107, and 451 nm for P3HT-O108 (***Fig. S10***).

3.2. Hydrogen Peroxide Quantification (HRP/TMB Method)

Hydrogen peroxide concentration was quantified spectrophotometrically using the horseradish peroxidase / 3,3′,5,5′-tetramethylbenzidine (HRP/TMB) oxidation method[32]. The assay is based on the HRP-catalyzed oxidation of TMB in the presence of H_2O_2. In this reaction, hydrogen peroxide acts as the oxidant, while HRP mediates the formation of reactive oxygen intermediates capable of converting TMB from its colorless reduced form to a blue oxidized chromophore (stable for pH 5.5–6.0). The oxidized TMB exhibits a characteristic absorption maximum at 653 nm. Aliquots ranging from 50 to 300 µL, depending on the hydrogen peroxide concentration in the sample, were combined with an appropriate volume of the HRP/TMB/PBS

mixture (prepared in accordance with the procedures outlined in subsections 3.2.1) so that the final volume in each case was 1000 µL. After 1–3 min, the absorbance at 653 nm was recorded. Concentrations were calculated from the calibration curve.

3.2.1. Preparation of Solutions for H_2O_2 Quantification

3.2.1.1. **Citrate–phosphate buffer** (pH 5.5 – 6.0)

To obtain a citrate–phosphate buffer in the desired pH range, 435 mL of the 0.2 M Na_2HPO_4 solution was mixed with 315 mL of 0.1 M citric acid. The pH was finely adjusted using small amounts of citric acid (to lower pH) or Na_2HPO_4 (to raise pH), yielding a final pH of 5.70.

3.2.1.2. **Preparation of the TMB solution** (10 mg/mL)

Tetramethylbenzidine (TMB) was dissolved in 25 mL of DMSO to obtain a 10 mg/mL stock solution. The solution was aliquoted into 1 mL microtubes, stored at −20°C, protected from light, and thawed only immediately prior to use.

3.2.1.3. **Preparation of the HRP solution** (2 mg/mL)

10 mg of horseradish peroxidase (HRP, type I, 90 U/mg) was dissolved in 5 ml DI water. The solution was aliquoted and stored at −20°C. A fresh portion was used for each measurement session to ensure reproducible enzymatic activity.

3.2.1.4. Preparation of the final reaction mixture for analysis

Immediately before analysis, the final reaction mixture was prepared. For a total volume of 10 mL, the components were combined in the following proportions: 9.93 mL of the 0.1 M citrate–phosphate buffer (pH 5.70), 20 µL of the HRP solution (2 mg/mL), and 50 µL of the TMB solution (10 mg/mL). The mixture was gently mixed and used within no more than 30 min after preparation.

3.3. Calibration curve for H_2O_2 Determination

A calibration curve was prepared using standard H_2O_2 solutions. A solution was prepared by diluting 40 µL of commercially available 30% H_2O_2 (9.80 M, confirmed through redox titration using a standardized solution of potassium permanganate in an acidic medium) to 1 L with DI water. Calibration solutions were prepared by pipetting 25 µL, 50 µL 75 µL, and 100 µL H_2O_2 stock solution into cuvettes and diluting with mixture of citrate-phosphate buffer containing the HRP/TMB reagent to a final volume of 1.00 mL in each case. After rapid mixing at room temperature, the absorbance at 653 nm was recorded. The calibration curve was prepared by plotting absorbance versus H_2O_2 concentration and applying linear regression according to:

$$A_{653} = a \cdot [H_2O_2] + b$$

The resulting coefficient of determination (R^2) was 0.997.

3.4. Parameter *k* determination

The efficiency of H_2O_2 evolution was evaluated in relation to the extent of material degradation using the parameter k. Parameter k was defined as the ratio of the number of moles of hydrogen

peroxide generated during the reaction ($n_{\mathrm{H_2O_2}}$) to the number of moles of P3HT repeat units initially used ($n_{\mathrm{cat,0}}$), either in the form of films or nanoparticles.

$$k = \frac{n_{H_2O_2}}{n_{cat,0}}$$

The amount of H_2O_2 formed was determined using the HRP/TMB method described above. The molar quantity of hydrogen peroxide in the reaction volume was calculated as:

$$n_{H_2O_2} = C_{H_2O_2} \cdot V_{sample}$$

where $C_{\mathrm{H_2O_2}}$ was obtained from the calibration curve and V_{sample} corresponds to the analyzed reaction volume.

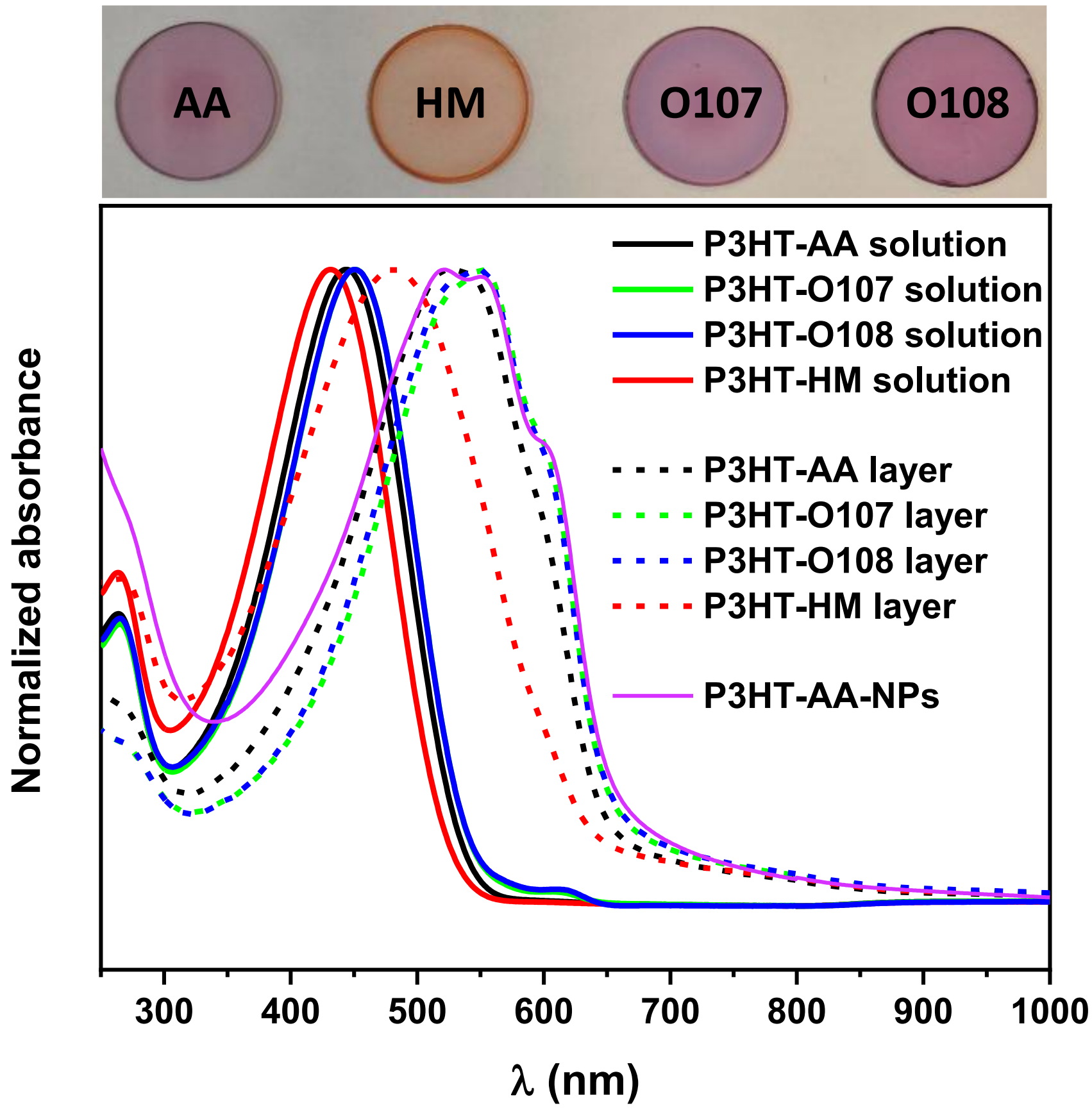

Fig. S8. UV–Vis spectra of P3HT in solution ($CHCl_3$), thin films deposited on quartz by spin coating, and nanoparticles (NPs) dispersed in water. Photographs of the corresponding spin-coated polymer films on quartz substrates are shown.

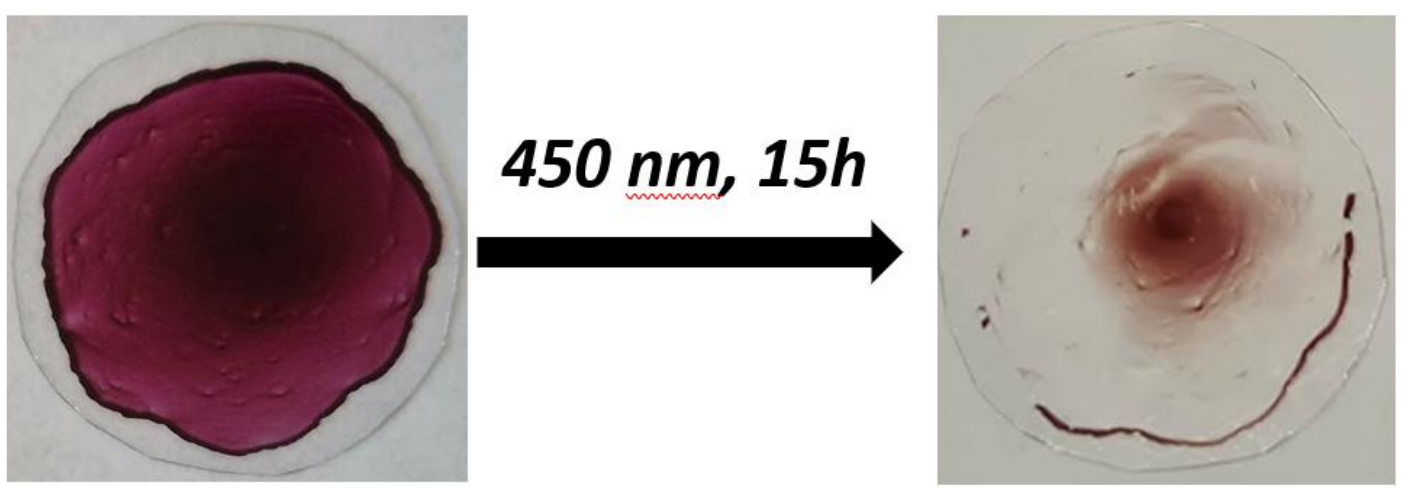

Fig. S9. Photographs of the P3HT-AA layers before and after 15 h of exposure to 450 nm light.

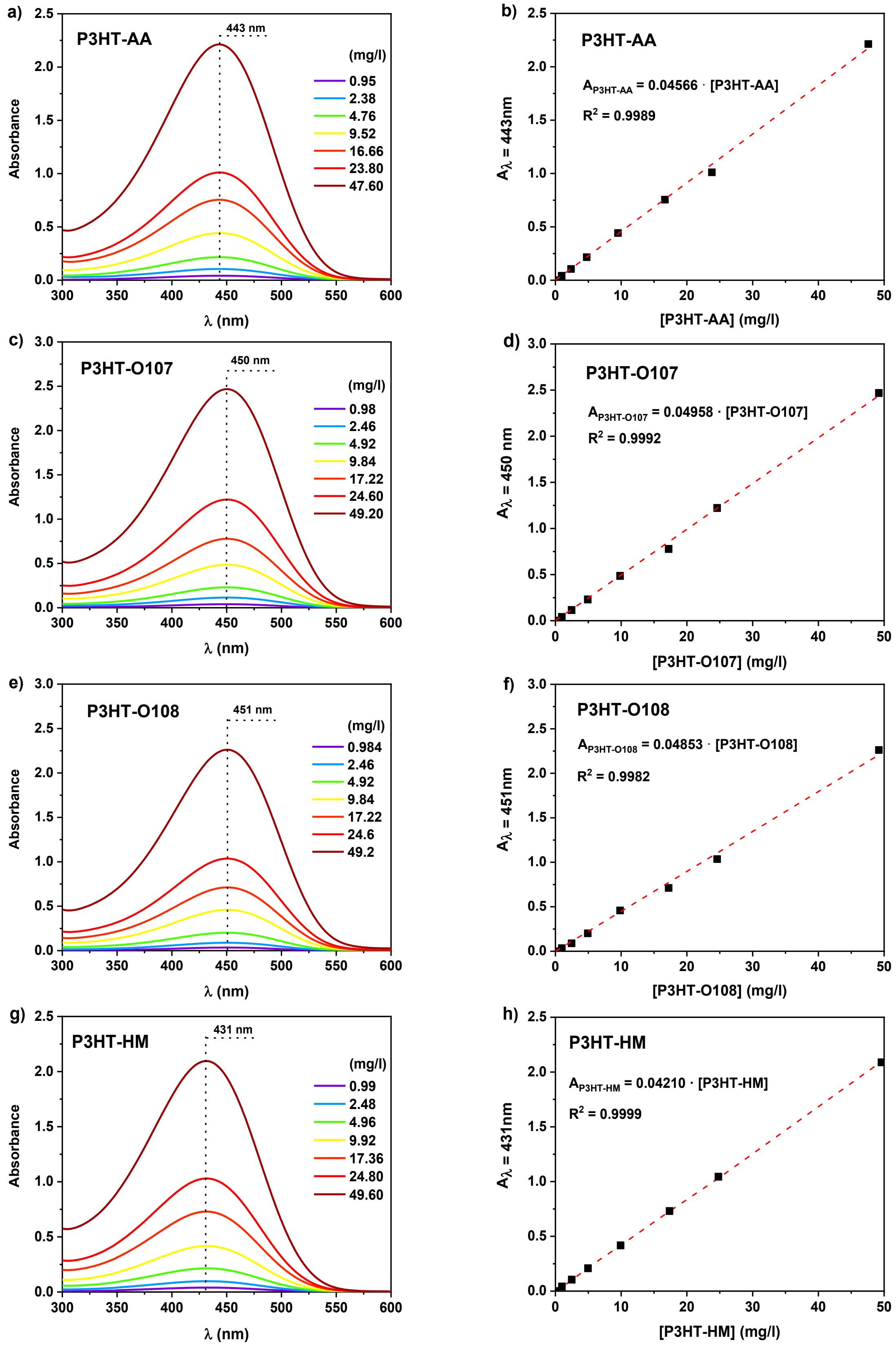

Fig. S10. UV–Vis spectra of the studied P3HT samples in chloroform (a, c, d, g) and the corresponding calibration plots (b, e, f, h). Optical path length L = 10.00 mm.

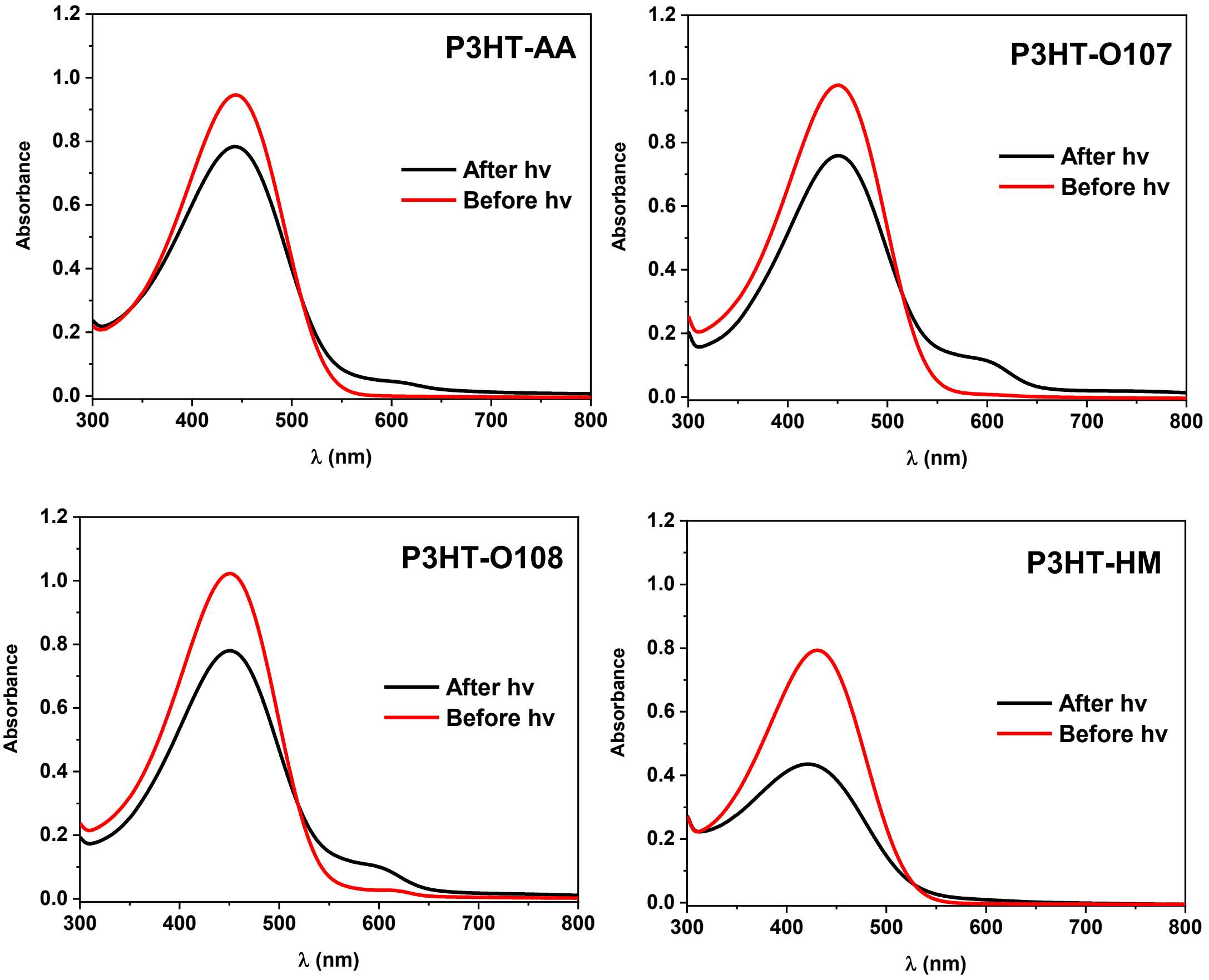

Figure S11. Representative UV-vis absorption spectra of P3HT-AA, P3HT-O107, P3HT-O108, and P3HT-HM solutions in DCM before and after 15 h irradiation at 523 nm in contact with HCOONa electrolyte. The spectra show a decrease in the intensity of the characteristic P3HT absorption band in the 350–550 nm range after irradiation, consistent with partial polymer degradation. These representative spectra supported the use of absorbance changes at the selected wavelengths to quantify the residual P3HT content in subsequent photodegradation experiments.

Table S2. Results of the irradiation experiment of AA-P3HT layers with different wavelengths. Each experiment was conducted for a 15h irradiation period using 300 ± 30 nmol of P3HT drop-casted as a layer on circular PET foils (Ø ≈ 1.5 cm). Thicknesses of the layers: d = 137 (±53) nm. The volume of the oxygenated solution was 0.4 mL. When used, the sacrificial donor was added at a concentration of 10 mM. The error values given in parentheses refer to the estimated standard deviation of four measurements performed on separate irradiated P3HT layers.

	**395 nm**			
	Water	***PBS***	***PBS+formate***	***PBS+glutathione***
Degradation / -	0.95 (0.01)	0.97 (0.02)	0.91 (0.02)	0.88 (0.01)
$[H_2O_2]$ / µM	560.4 (23.5)	549.1 (32.7)	537.8 (29.8)	
k / $n_{H2O2}/n_{cat,0}$	0.757 (0.026)	0.841 (0.050)	0.661 (0.037)	
	**457 nm**			
	Water	***PBS***	***PBS+formate***	***PBS+glutathione***
Degradation / -	0.51 (0.04)	0.52 (0.06)	0.51 (0.05)	0.35 (0.08)
$[H_2O_2]$ / µM	167.5 (18.5)	164.8 (28.2)	168.1 (12.4)	
k / $n_{H2O2}/n_{cat,0}$	0.204 (0.023)	0.206 (0.035)	0.199 (0.015)	
	**500 nm**			
	Water	***PBS***	***PBS+formate***	***PBS+glutathione***
Degradation / -	0.19 (0.05)	0.22 (0.05)	0.23 (0.04)	0.21 (0.03)
$[H_2O_2]$ / µM	37.2 (2.9)	34.4 (5.3)	27.5 (4.5)	
k / $n_{H2O2}/n_{cat,0}$	0.049 (0.004)	0.043 (0.007)	0.037 (0.006)	
	**523 nm**			
	Water	***PBS***	***PBS+formate***	***PBS+glutathione***
Degradation / -	0.14 (0.03)	0.23 (0.05)	0.23 (0.06)	0.17 (0.05)
$[H_2O_2]$ / µM	14.0 (1.9)	22.8 (2.7)	25.6 (3.0)	
k / $n_{H2O2}/n_{cat,0}$	0.020 (0.003)	0.031 (0.004)	0.034 (0.004)	
	**625 nm**			
	Water	***PBS***	***PBS+formate***	***PBS+glutathione***
Degradation / -	0.08 (0.03)	0.09 (0.02)	0.11 (0.02)	0.07 (0.01)
$[H_2O_2]$ / µM	6.0 (0.6)	8.6 (0.7)	9.9 (0.9)	
k / $n_{H2O2}/n_{cat,0}$	0.009 (0.001)	0.014 (0.001)	0.012 (0.001)	
	**dark**			
	Water	***PBS***	***PBS+formate***	***PBS+glutathione***
Degradation / -	0.03 (0.03)	0.03 (0.01)	0.00 (0.03)	0.00 (0.02)
$[H_2O_2]$ / µM	0.0 (0.0)	0.0 (0.0)	0.0 (0.0)	
k / $n_{H2O2}/n_{cat,0}$	0.000 (0.000)	0.000 (0.000)	0.000 (0.000)	

Table S3. Results of the irradiation experiment of P3HT layers prepared from polymers supplied by different vendors and of nanoparticles obtained from the P3HT-AA polymer. Each experiment was conducted for 15 h under irradiation at a wavelength of 523 nm. For the layers, 300 ± 30 nmol of P3HT was deposited by drop-casting onto circular PET foils and immersed in 0.4 mL of oxygenated PBS solution with formate (0.1 mM). For the nanoparticles, 45 nmol of P3HT was used as a dispersion in 0.4 mL of PBS (without NaCl and KCl) containing formate (10 mM). The error values given in parentheses refer to the estimated standard deviation of four measurements performed on separate irradiated P3HT layers. Total surface areas were estimated using simplified geometric assumptions. For P3HT nanoparticles, spherical and non-porous particles were assumed, with the number-average diameter determined by DLS (177 nm) and a P3HT density of 1.1 g cm^{-3}. The specific surface area was calculated from ($S/m = 6/\rho \cdot d$), giving approximately 30.8 m^2g^{-1}. Based on the amount of P3HT used in the irradiation experiment (45 nmol of repeat units, corresponding to 7.48 µg of polymer in 0.4 ml aqueous solution), the total nanoparticle surface area was estimated to be approximately 2.3 cm^2. For planar P3HT films, the exposed surface area was taken as the geometric area of one side of the 15 mm diameter PET disc, corresponding to approximately 1.77 cm^2.

	P3HT-AA	***P3HT-O107***	***P3HT-O108***	***P3HT-HM***	***P3HT-AA-NP***
Total surface area / cm²	~1.77				~2.3
Degradation / -	0.23 (0.06)	0.23 (0.04)	0.20 (0.06)	0.47 (0.03)	0.76 (0.03)
$[H_2O_2]$ / µM	25.6 (3.0)	21.7 (3.0)	24.5 (1.0)	74.1 (2.5)	38.1 (1.3)
k / $n_{H2O2}/n_{cat,0}$	0.034 (0.004)	0.030 (0.008)	0.030 (0.001)	0.108 (0.004)	0.420 (0.015)

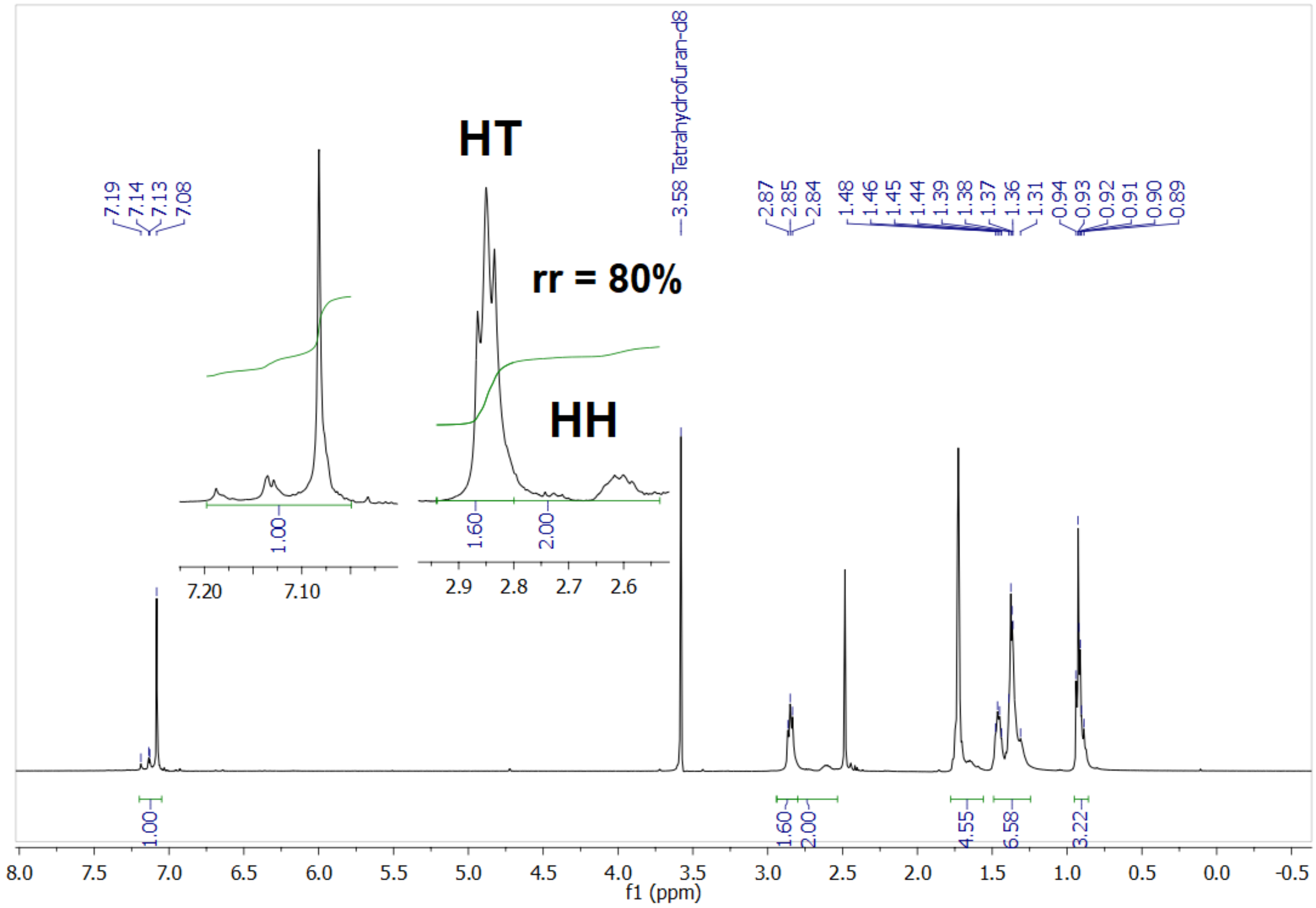

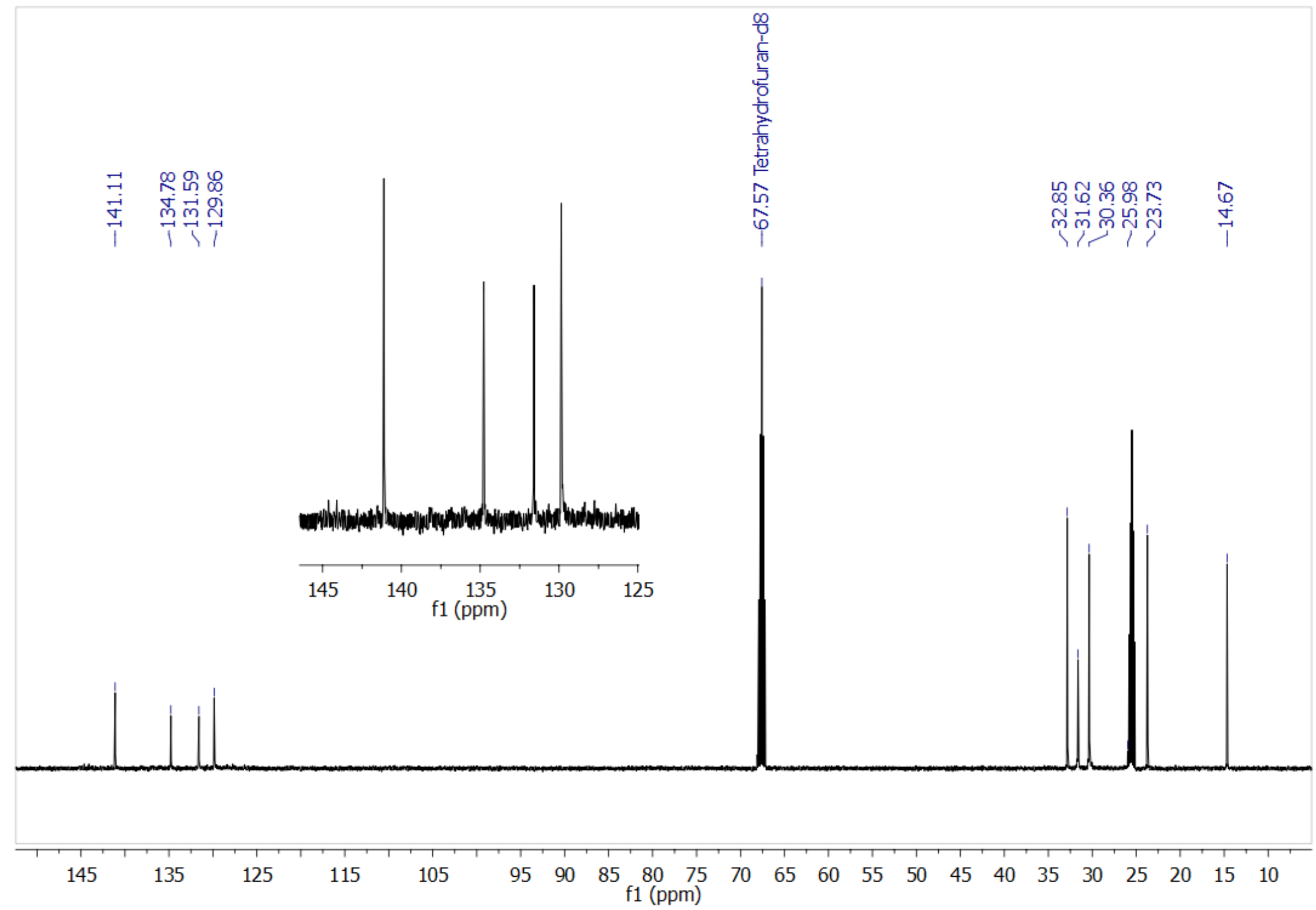

Fig. S12. ^{1}H and ^{13}C NMR spectra of **P3HT-AA** in THF-d8.

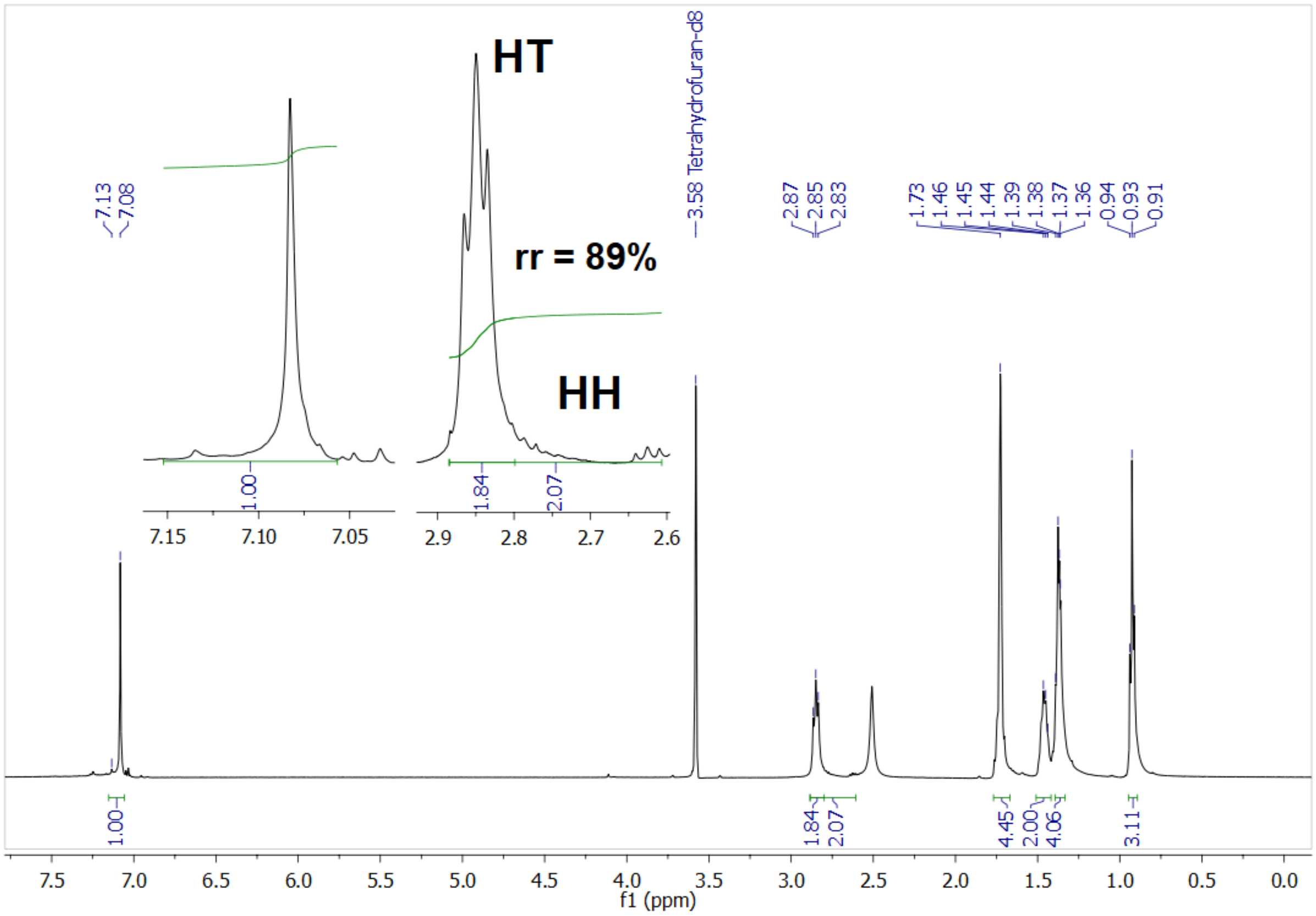

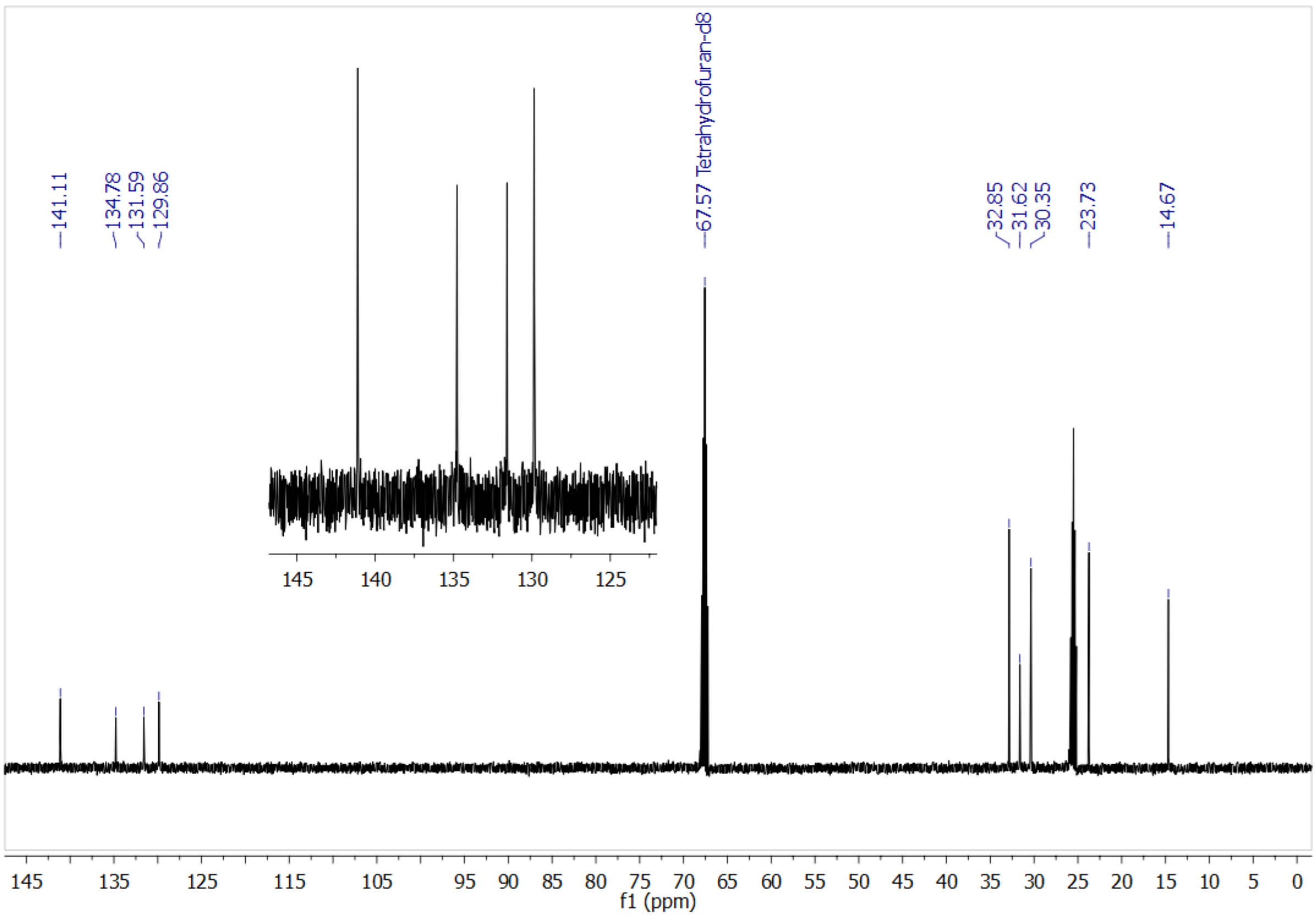

Fig. S13. ^{1}H and ^{13}C NMR spectra of **P3HT-O107** in THF-d8.

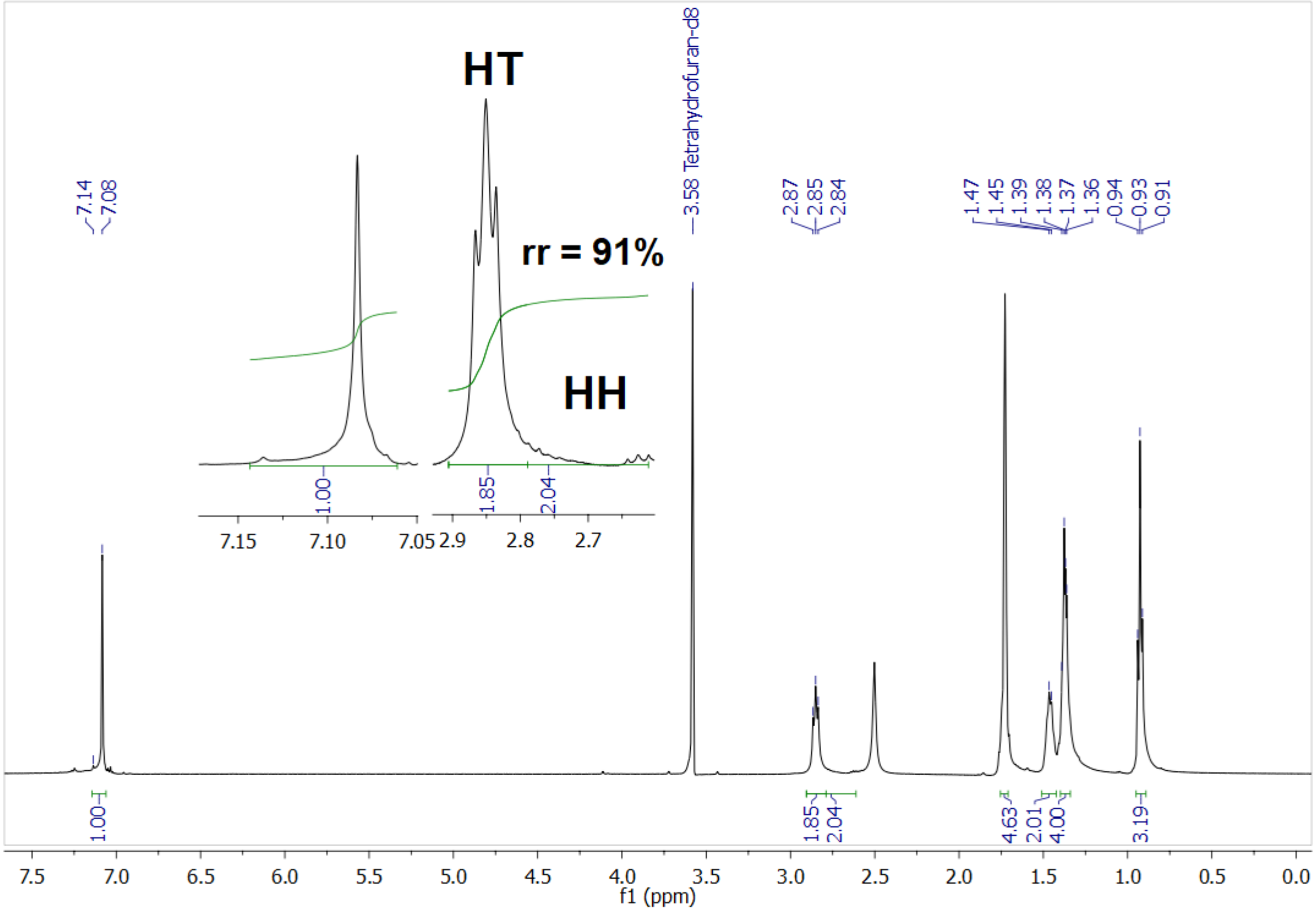

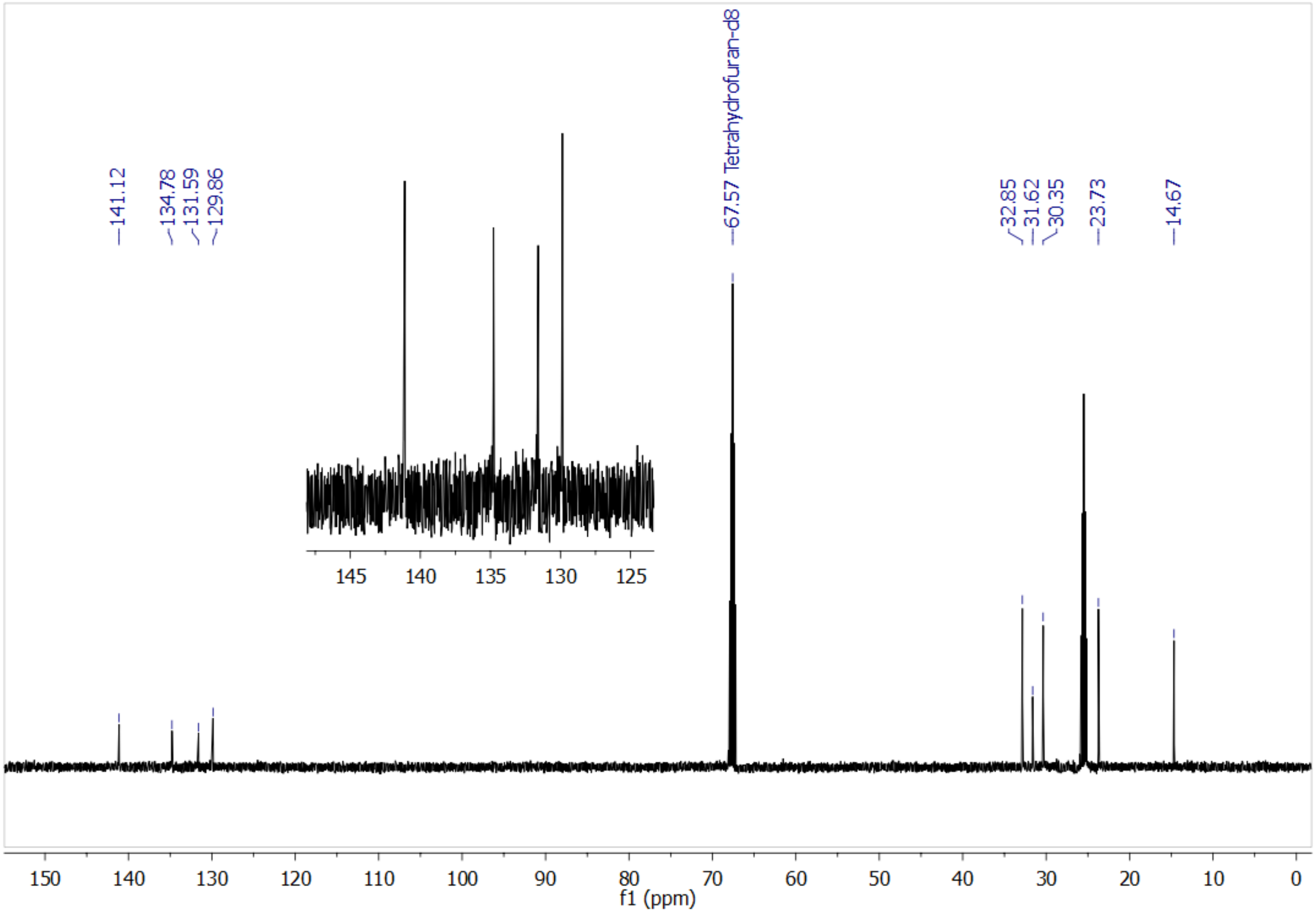

Fig. S14. ^{1}H and ^{13}C NMR spectra of **P3HT-O108** in THF-d8.

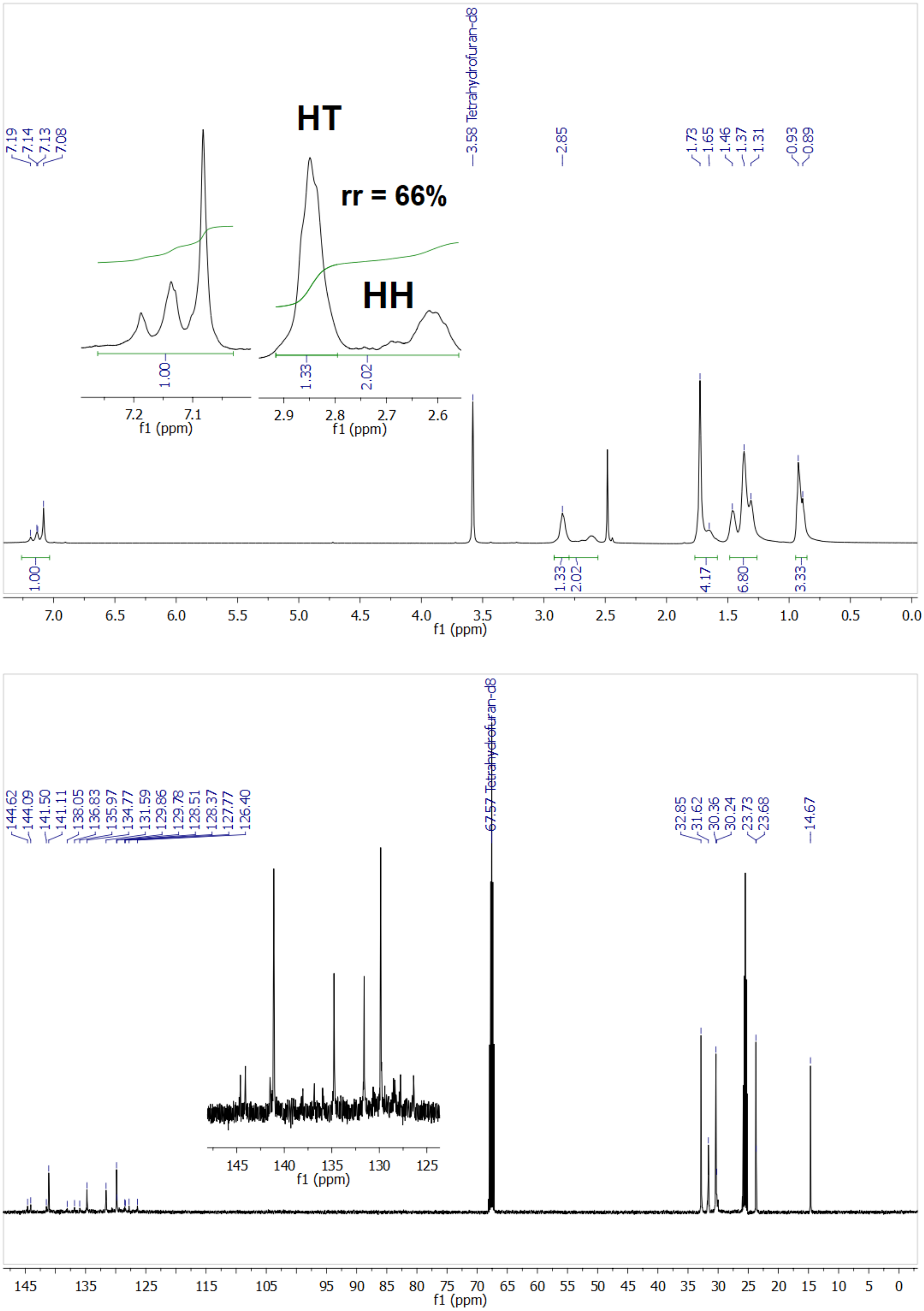

Fig. S15. ^{1}H and ^{13}C NMR spectra of **P3HT-HM** in THF-d8.

4. Suplementary references

[4] J. F. Maya-Vetencourt, G. Manfredi, M. Mete, E. Colombo, M. Bramini, S. Di Marco, D. Shmal, G. Mantero, M. Dipalo, A. Rocchi, M. L. DiFrancesco, E. D. Papaleo, A. Russo, J. Barsotti, C. Eleftheriou, F. Di Maria, V. Cossu, F. Piazza, L. Emionite, F. Ticconi, C. Marini, G. Sambuceti, G. Pertile, G. Lanzani, F. Benfenati, "Subretinally injected semiconducting polymer nanoparticles rescue vision in a rat model of retinal dystrophy" *Nat. Nanotechnol.* **2020**, *15*, 698–708.

[5] C. Tortiglione, M. R. Antognazza, A. Tino, C. Bossio, V. Marchesano, A. Bauduin, M. Zangoli, S. V. Morata, G. Lanzani, "Semiconducting polymers are light nanotransducers in eyeless animals" *Sci. Adv.* **2017**, *3*, e1601699.

[12] E. Zucchetti, M. Zangoli, I. Bargigia, C. Bossio, F. Di Maria, G. Barbarella, C. D'Andrea, G. Lanzani, M. R. Antognazza, "Poly(3-hexylthiophene) nanoparticles for biophotonics: study of the mutual interaction with living cells" *J. Mater. Chem. B* **2017**, *5*, 565–574.

[28] G. Barbarella, A. Bongini, M. Zambianchi, "Regiochemistry and Conformation of Poly(3-hexylthiophene) via the Synthesis and the Spectroscopic Characterization of the Model Configurational Triads" *Macromolecules* **1994**, 27, 3039–3045

[29] D. Tuncel, H. V. Demir, "Conjugated polymer nanoparticles" *Nanoscale* **2010**, *2*, 484.

[30] J. Pecher, S. Mecking, "Nanoparticles of Conjugated Polymers" *Chem. Rev.* **2010**, *110*, 6260–6279.

[31] H. Shimizu, M. Yamada, R. Wada, M. Okabe, "Preparation and Characterization of Water Self-dispersible Poly(3-hexylthiophene) Particles" *Polym. J.* **2008**, *40*, 33–36.

[32] M. Gryszel, A. Markov, M. Vagin, E. D. Głowacki, "Organic heterojunction photocathodes for optimized photoelectrochemical hydrogen peroxide production" *J. Mater. Chem. A* **2018**, *6*, 24709–24716.
